# Supplementary material for: Structure of the catalytic core of the Integrator complex
Source: Mol Cell. 2021 Mar 18;81(6):1246–1259.e8. doi: 10.1016/j.molcel.2021.01.005 (PMC7980224; doi:10.1016/j.molcel.2021.01.005)
Supplement: Document S1. Figures S1–S7 [file mmc1.pdf]

Molecular Cell, Volume *81*

Supplemental information

Structure of the catalytic core of the Integrator complex

Moritz M. Pfleiderer and Wojciech P. Galej

SUPPLEMENTARY INFORMATION

Figure S1

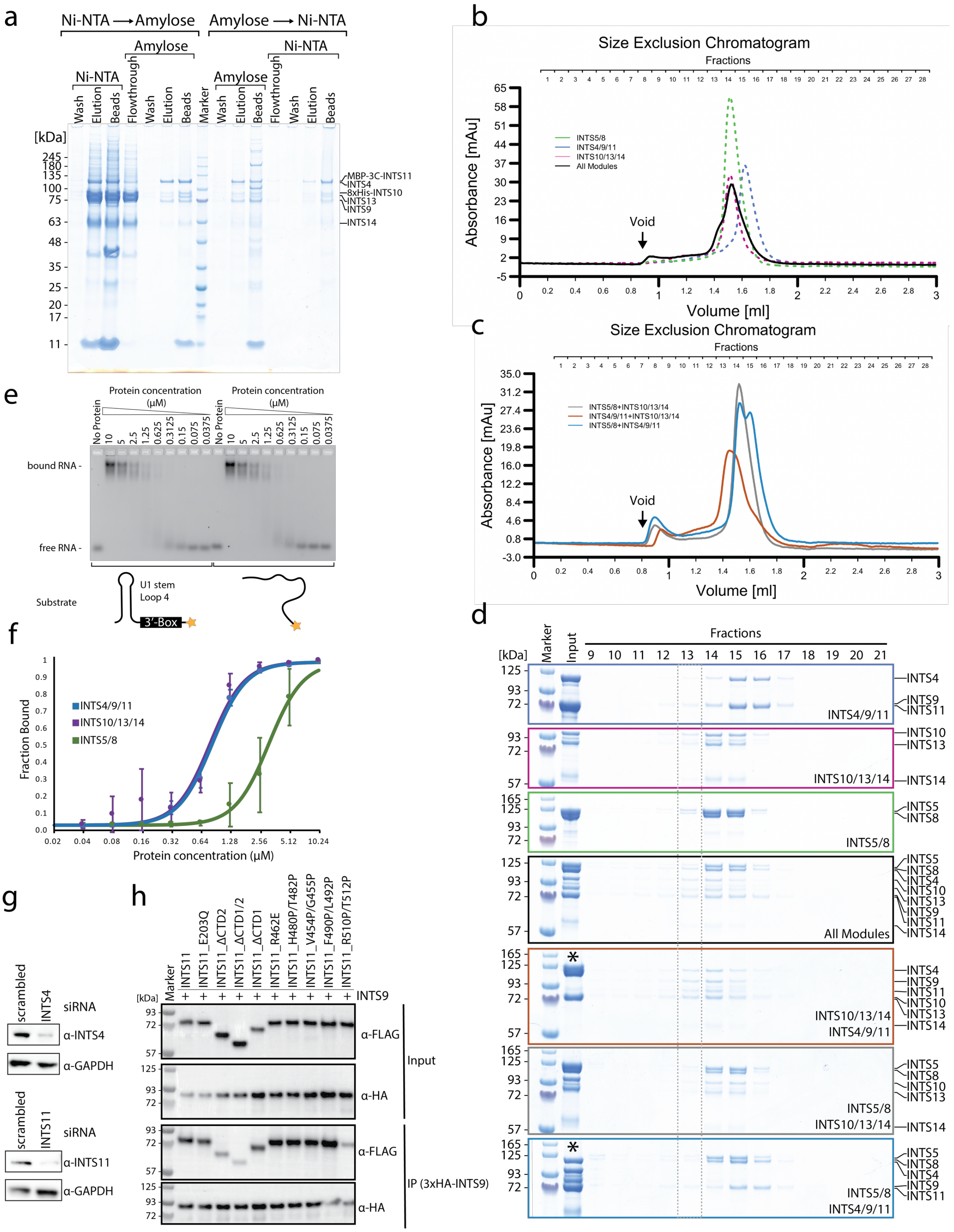

Figure S1. Higher order complex formation by INTS4/9/11 and INTS10/13/14 and their RNA-binding properties. Related to Figures 1,3,4 and 6.

a, SDS-PAGE showing the results of recombinant expression and tandem affinity purification of all 6 Integrator subunits forming INTS4/9/11 and INTS10/13/14 modules. MBP tag was fused to INTS11 and 6xHistag was attached to INTS10. **b,** SEC profile showing the elution volume of INTS5/8, INTS4/9/11 and INTS10/13/14 as single complexes (dotted lines) and after being mixed together (black line). **c,** SEC profiles of the different sub-complexes mixed together to test their interactions with one another. **d,** SDS-PAGE of the different fractions corresponding to the peaks of the SECs in panel b and c. Dotted line indicates fractions containing the higher order complex. Two lanes marked with asterisks (*) were accidentally swapped during SDS-PAGE loading. **e,** EMSA titration experiment, in which all 3 modules were used to assess their RNA binding. No clear cooperativity was observed when compared to individual titrations (Figure 6) **f,** Semi-quantitative RNA-binding analysis based on EMSA experiments performed in triplicates. The titration curves report the fraction of RNA bound ([RNP]/[RNA]+[RNP]) against protein concentration (logarithmic scale). The estimated apparent Kd's are as follows: 0.82±0.06 μM (INTS4/9/11) 3.1±1 μM (INTS5/8) and 0.78±0.08 μM (INTS10/13/14). The uncertainty of the apparent Kd was estimated based on standard deviation of the nearest experimental titration point. **g,** Western blots showing efficiency of the siRNA knock-down experiment for INTS4 and INTS11. **h,** Western blot showing results of the HA-agarose pull-down experiments from HEK293T cells co-expressing 3xHA-INTS9 and 3xFLAG-INTS11 variants.

Figure S2

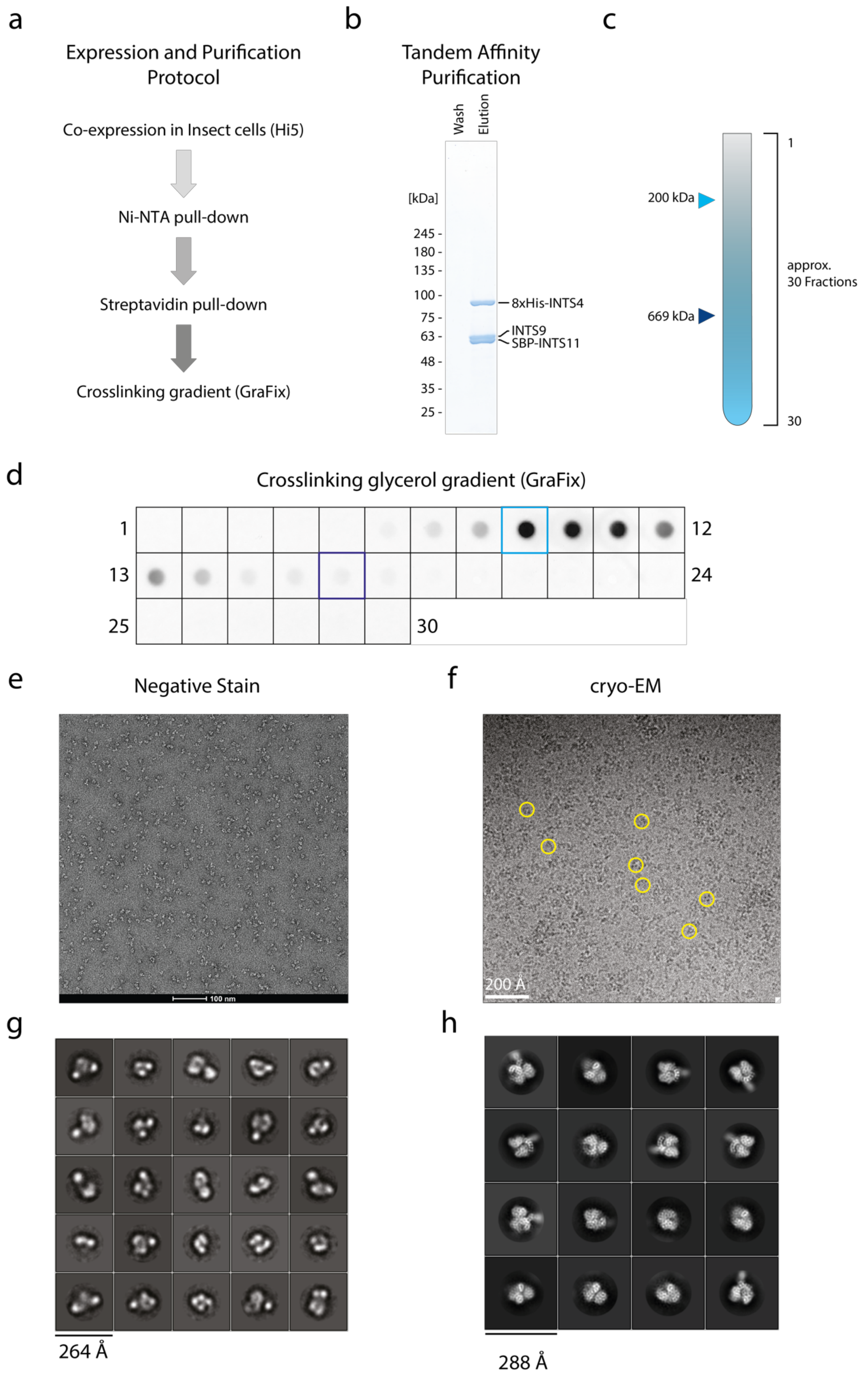
a
Expression and Purification Protocol
Co-expression in Insect cells (Hi5)
Ni-NTA pull-down
Streptavidin pull-down
Crosslinking gradient (GraFix)
b
Tandem Affinity Purification
Wash
Elution
[kDa]
245 -
180 -
135 -
100 -
75 -
63 -
48 -
35 -
25 -
8xHis-INTS4
INTS9
SBP-INTS11
c
200 kDa
669 kDa
1
approx. 30 Fractions
30
d
Crosslinking glycerol gradient (GraFix)
1
12
13
24
25
30
e
Negative Stain
100 nm
f
cryo-EM
200 Å
g
264 Å
h
288 Å

Figure S2. Sample preparation and initial cryo-EM analysis. Related to Figure 2.

a, Flow chart of the expression and purification of the INTS4/9/11 complex. **b,** SDS-PAGE after the two-step purification indicated in panel a. **c** and **d,** Illustration of a calibrated cross-linking gradient and Dot-blot analysis of the different GraFix fractions with anti-SBP antibodies to detect the migration of the INTS4/9/11 complex. **e,** representative uranyl acetate negative stain micrograph. **f,** representative cryo-EM micrograph from a 300kV Titan Krios Microscope and K2 detector at -2.85 μm defocus. Examples of particles picked for processing are indicated with yellow circles. **g,** reference-free 2D class averages of a small negative stain dataset. **h,** reference-free 2D class averages of the cryo-EM dataset.

Figure S3

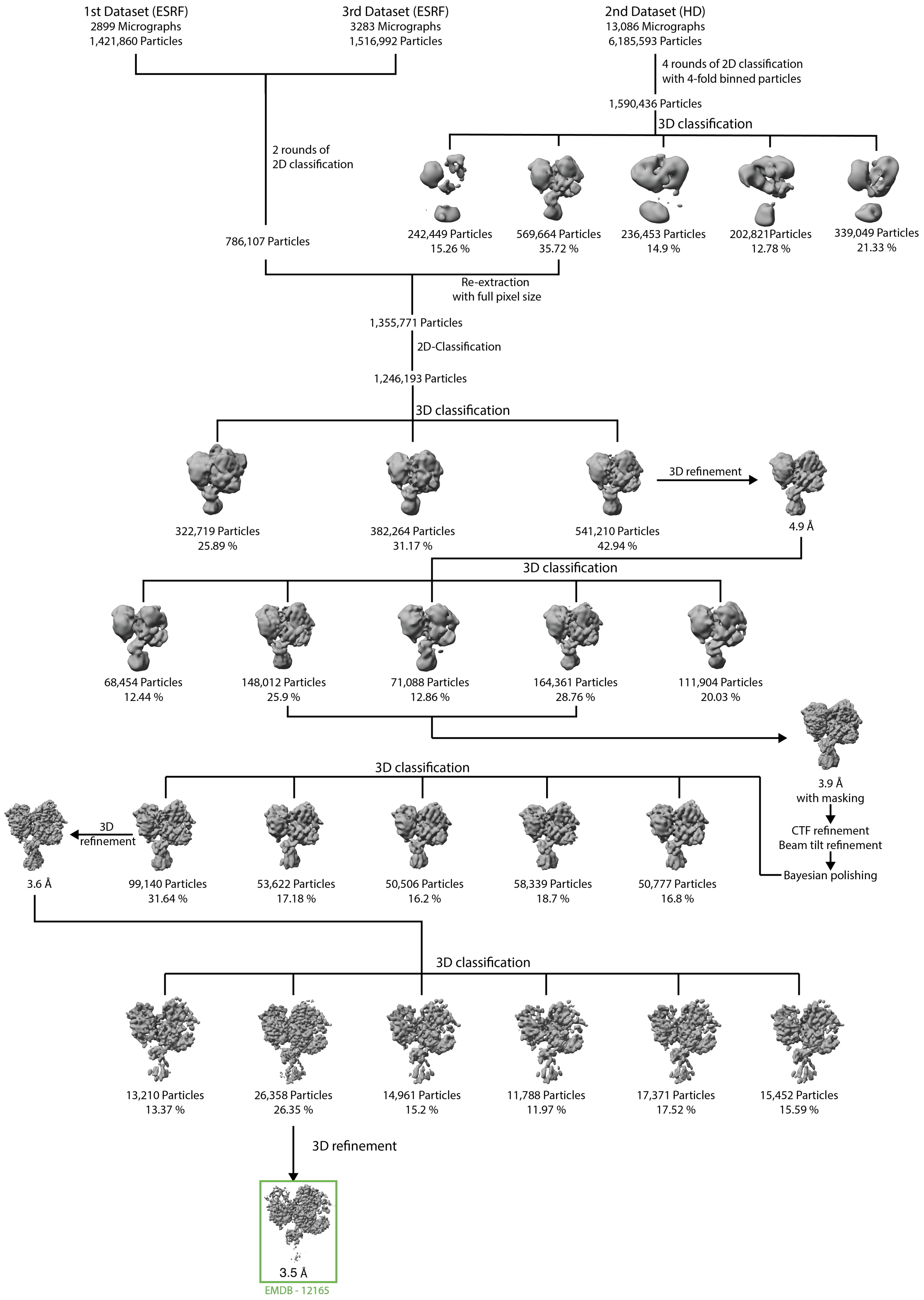

Figure S3. Workflow for the cryo-EM data processing. Related to Figure 2 and the STAR method section: Cryo-EM data collection and processing.

Three datasets were merged and processed as described in details in the method section.

Figure S4

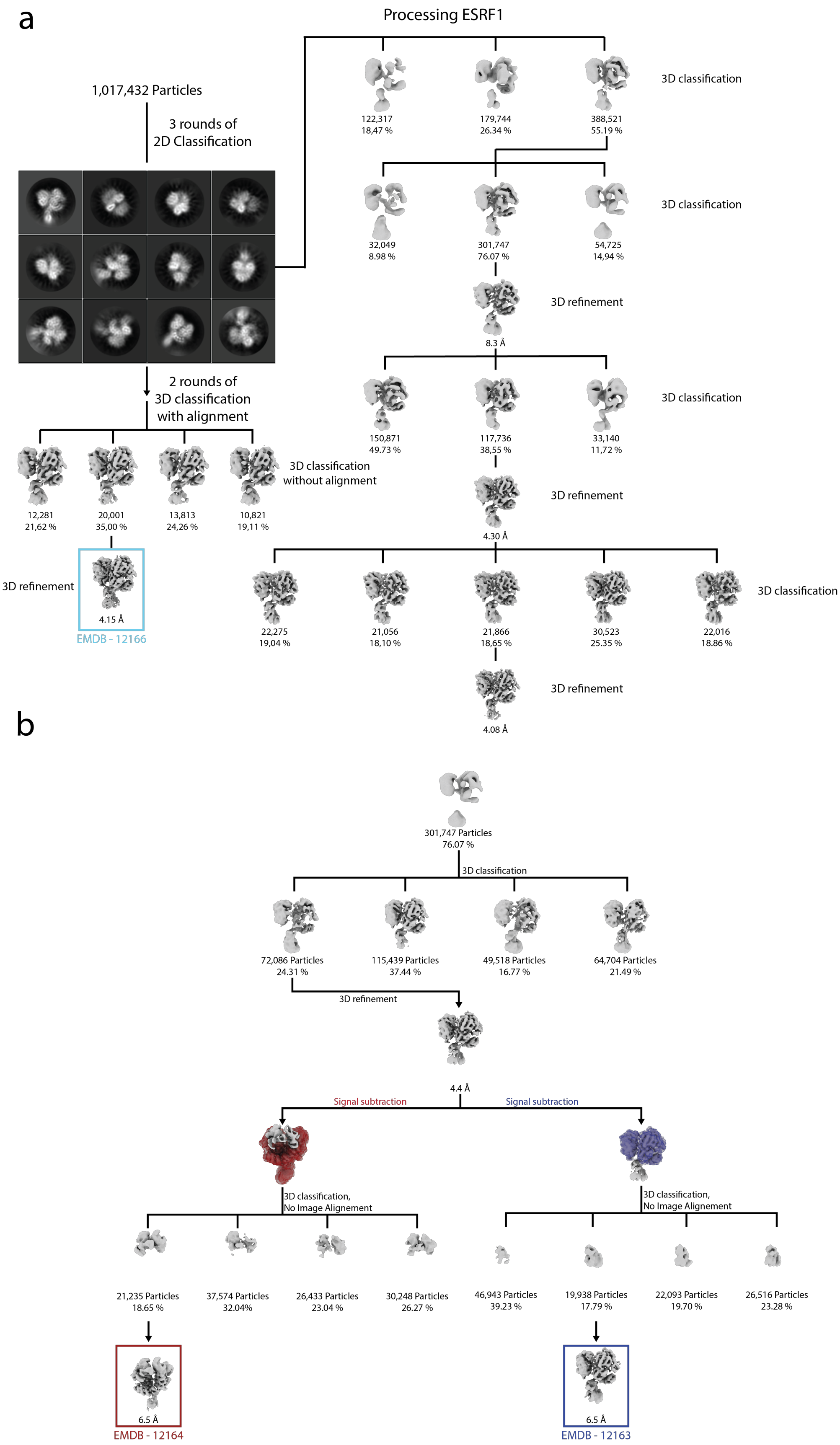
a
Processing ESRF1
1,017,432 Particles
3 rounds of
2D Classification
3D classification
122,317
18,47 %
179,744
26.34 %
388,521
55.19 %
32,049
8.98 %
301,747
76.07 %
54,725
14,94 %
3D refinement
8.3 Å
150,871
49.73 %
117,736
38,55 %
33,140
11,72 %
4.30 Å
2 rounds of
3D classification
with alignment
3D classification
without alignment
12,281
21,62 %
20,001
35,00 %
13,813
24,26 %
10,821
19,11 %
4.15 Å
EMDB - 12166
22,275
19,04 %
21,056
18,10 %
21,866
18,65 %
30,523
25.35 %
22,016
18.86 %
4.08 Å
b
301,747 Particles
76.07 %
72,086 Particles
24.31 %
115,439 Particles
37.44 %
49,518 Particles
16.77 %
64,704 Particles
21.49 %
4.4 Å
Signal subtraction
3D classification,
No Image Alignement
21,235 Particles
18.65 %
37,574 Particles
32.04%
26,433 Particles
23.04 %
30,248 Particles
26.27 %
46,943 Particles
39.23 %
19,938 Particles
17.79 %
22,093 Particles
19.70 %
26,516 Particles
23.28 %
6.5 Å
EMDB - 12164
EMDB - 12163

Figure S4. Workflow for the focused classification and refinements used in this study. Related to Figure 2 and the STAR method section: Cryo-EM data collection and processing.

a, processing flowchart of the ESRF1 dataset contining peripheral regions of the complex. **b,** focussed classification and refinement workflow for $INTS4^{NTD}$ (red) and CTD2 (blue), based on ESRF1 dataset.

Figure S5

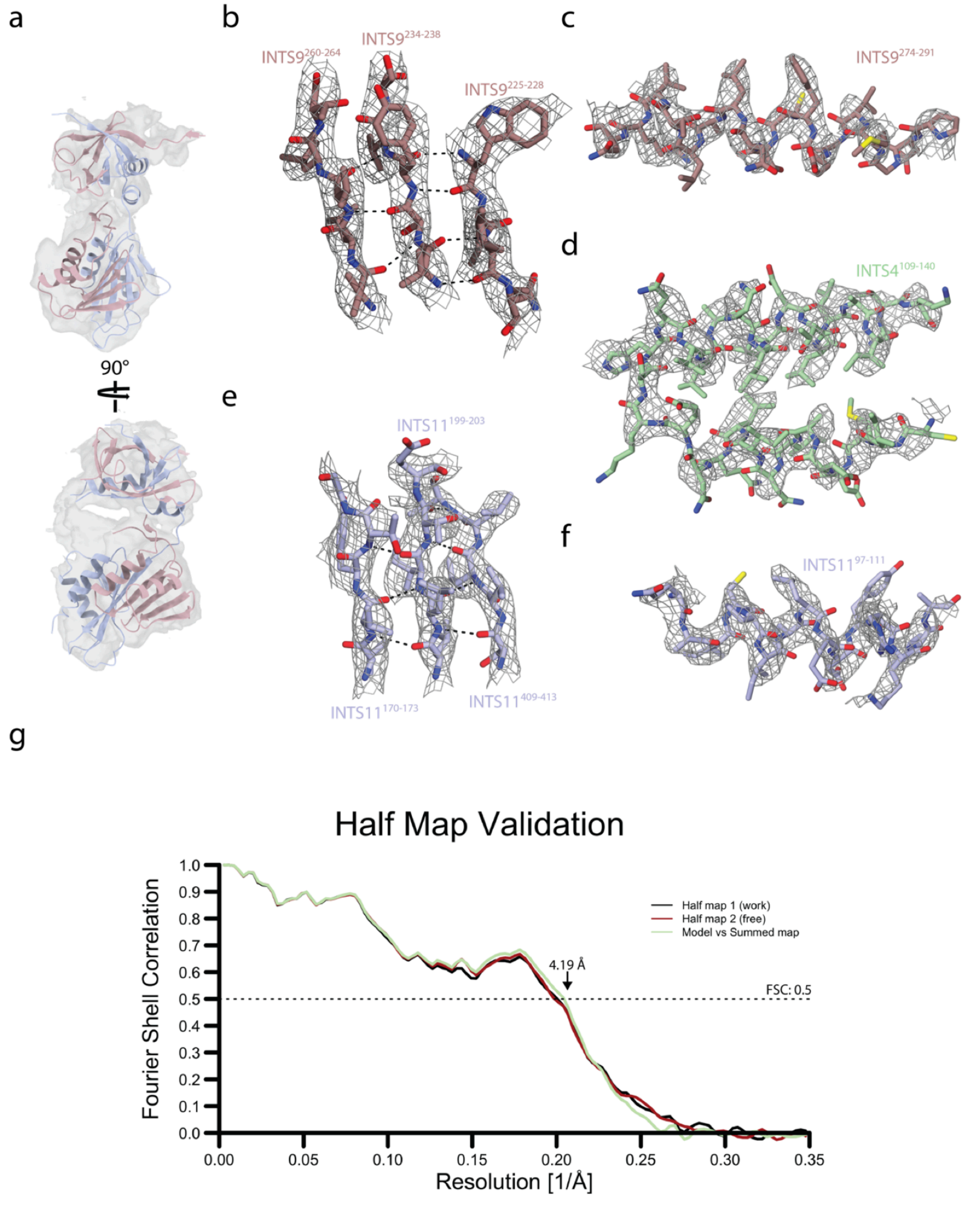

Figure S5. Model building of INTS4/9/11. Related to Figure 2.

a, INTS9/11^{CTD2}-focused map with the fitted crystal structure (PDB: 5V8W) and the de-novo modelled CTD1. **b-f,** Density at 3.5 Å with the built model of INTS4/9/11 in different areas of the map. **g,** Half map validation plot.

Figure S6

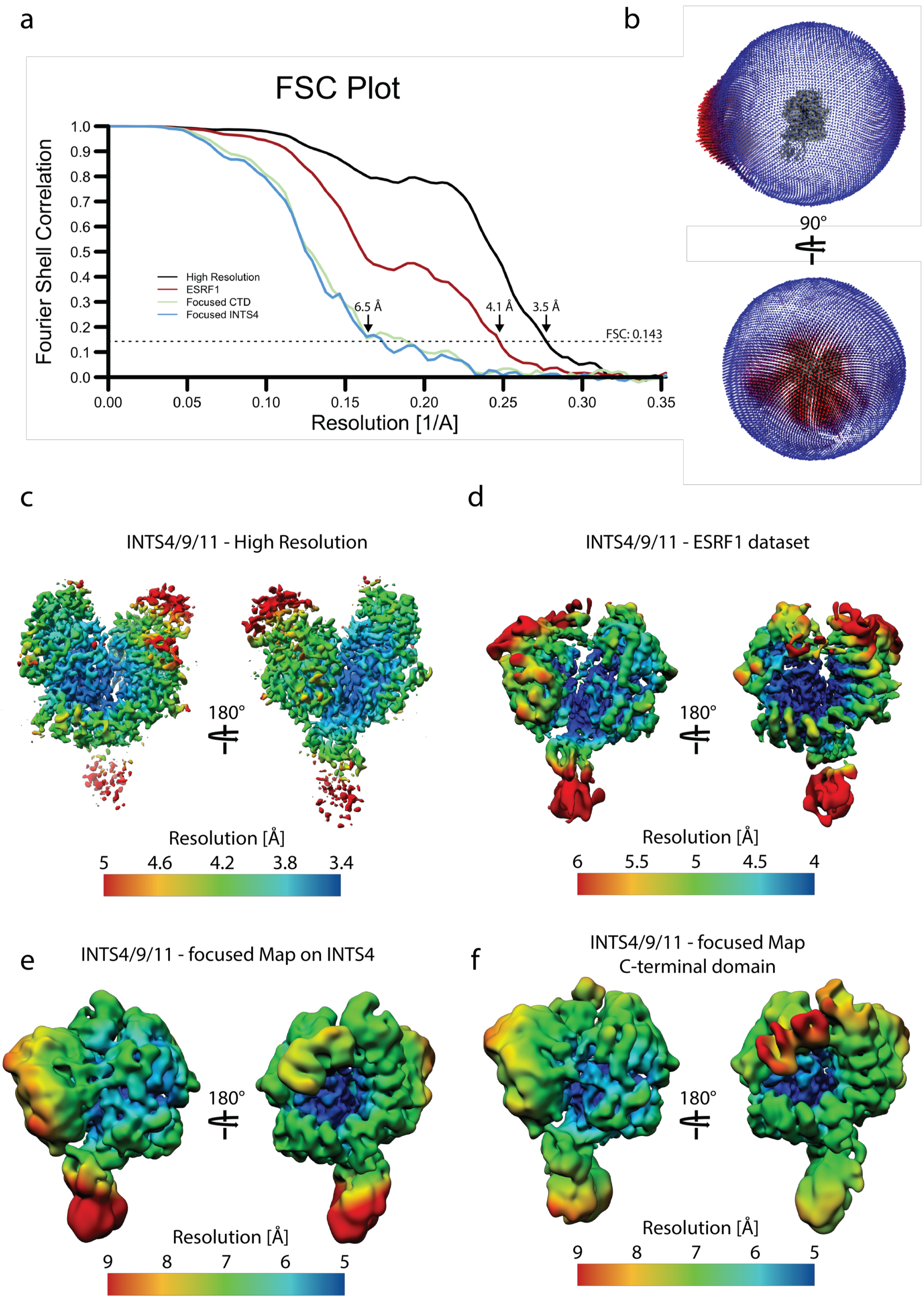

Figure S6. Global and local resolution of the different INTS4/9/11 maps generated in this study. Related to Figures 2, 3 and 4.

a, FSC plot for all four models used in this publication. **b,** Orientation distribution of the final, 3.5 Å map used for model building. **c-f,** cryo-EM maps from different reconstructions, coloured according to the local resolution.

Figure S7

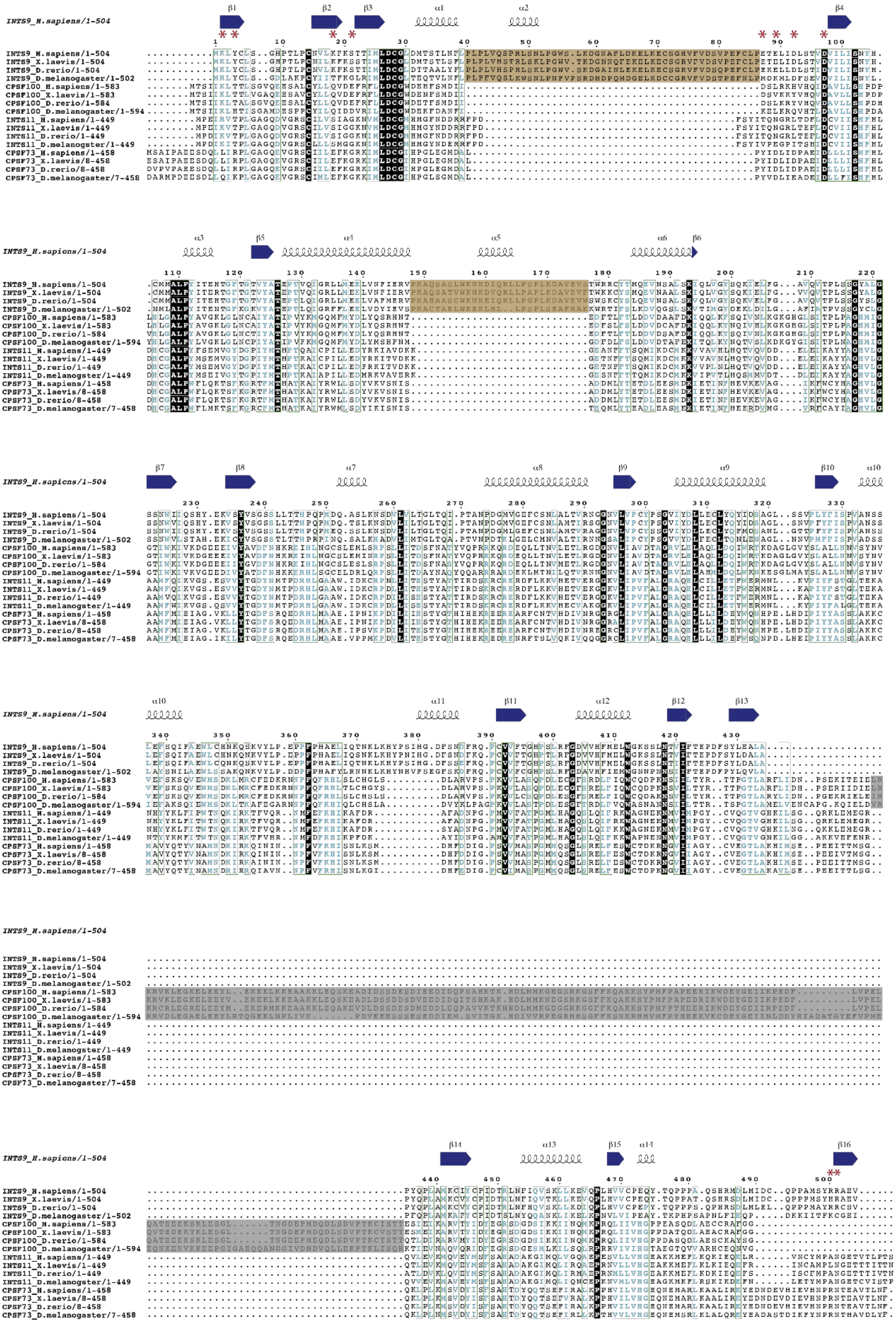
INTS9_H.sapiens/1-504
INTS9_X.laevis/1-504
INTS9_D.rerio/1-504
INTS9_D.melanogaster/1-502
CPSF100_H.sapiens/1-583
CPSF100_X.laevis/1-583
CPSF100_D.rerio/1-584
CPSF100_D.melanogaster/1-594
INTS11_H.sapiens/1-449
INTS11_X.laevis/1-449
INTS11_D.rerio/1-449
INTS11_D.melanogster/1-449
CPSF73_H.sapiens/1-458
CPSF73_X.laevis/8-458
CPSF73_D.rerio/8-458
CPSF73_D.melanogaster/7-458

Figure S7. Comparison of the nuclease domains of INTS9, INTS11, CPSF73 and CPSF100. Related to Figures 2 and 5.

Structure-based alignment of INTS9, INTS11 CPSF73 and CPSF100 of different organisms (human - *H. sapiens*; African clawed frog – *X. laevis*; Zebrafish – *D. rerio*; Fruit fly – *D. melanogaster*). The NAD domain of INTS9 is highlighted in yellow and the disordered insert of CPSF100, which contains the PIM, is coloured in grey. Asterisks indicate the residues of the active centre of INTS11. Black background – strictly identical; light blue – conserved residues; green frame – conserved area.
