# Supplementary material for: Structure of the catalytic core of the Integrator complex
Source: Mol Cell. 2021 Mar 18;81(6):1246–1259.e8. doi: 10.1016/j.molcel.2021.01.005 (PMC7980224; doi:10.1016/j.molcel.2021.01.005)
Supplement: Document S2. Article plus supplemental information [file mmc4.pdf]

Molecular Cell

Article

Structure of the catalytic core of the Integrator complex

Graphical Abstract

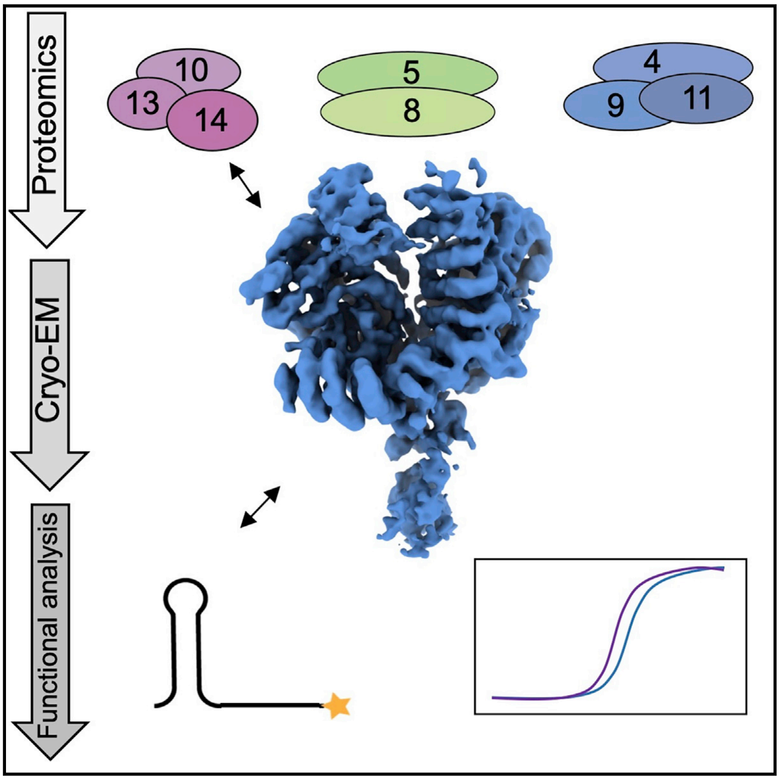

Authors

Moritz M. Pfleiderer, Wojciech P. Galej

Correspondence

wgalej@embl.fr

In Brief

Pfleiderer and Galej performed biochemical and structural analysis of the Integrator complex, one of the three main 3′ end-processing machineries in higher eukaryotes. The study reveals the modular nature of the Integrator complex, and the cryo-EM structure of its catalytic core provides detailed insights into its architecture and assembly mechanism.

Highlights

- Native Integrator complex is highly modular
- INTS5/8, INTS10/13/14, and INTS4/9/11 exist as stable sub-complexes
- Cryo-EM structure of the INTS4/9/11 reveals a putative substrate binding grove
- CTD1 of INTS9/11 is required for recruitment of INTS4

Pfleiderer & Galej, 2021, Molecular Cell *81*, 1246–1259
March 18, 2021 © 2021 The Author(s). Published by Elsevier Inc.
https://doi.org/10.1016/j.molcel.2021.01.005

CellPress
OPEN ACCESS

Molecular Cell

Article

Structure of the catalytic core of the Integrator complex

Moritz M. Pfleiderer[1] and Wojciech P. Galej[1,2,*]
[1]European Molecular Biology Laboratory, 71 Avenue des Martyrs, 38042 Grenoble, France
[2]Lead contact
*Correspondence: wgalej@embl.fr
https://doi.org/10.1016/j.molcel.2021.01.005

SUMMARY

The Integrator is a specialized 3′ end-processing complex involved in cleavage and transcription termination of a subset of nascent RNA polymerase II transcripts, including small nuclear RNAs (snRNAs). We provide evidence of the modular nature of the Integrator complex by biochemically characterizing its two subcomplexes, INTS5/8 and INTS10/13/14. Using cryoelectron microscopy (cryo-EM), we determined a 3.5-Å-resolution structure of the INTS4/9/11 ternary complex, which constitutes Integrator's catalytic core. Our structure reveals the spatial organization of the catalytic nuclease INTS11, bound to its catalytically impaired homolog INTS9 via several interdependent interfaces. INTS4, a helical repeat protein, plays a key role in stabilizing nuclease domains and other components. In this assembly, all three proteins form a composite electropositive groove, suggesting a putative RNA binding path within the complex. Comparison with other 3′ end-processing machineries points to distinct features and a unique architecture of the Integrator's catalytic module.

INTRODUCTION

3′ end processing of nascent RNA polymerase II (RNAPII) transcripts is one of the key steps in gene expression (Proudfoot, 2011; Shi and Manley, 2015). Nearly all protein-coding RNAPII transcripts are cleaved and polyadenylated by the cleavage and polyadenylation specificity factor (CPSF) (Bienroth et al., 1993; Murthy and Manley, 1992; Preker et al., 1997). The exceptions include replication-dependent histone pre-mRNAs, which are processed by a partially overlapping machinery that depends on the U7 small nuclear ribonucleoprotein particle (snRNP) (Mowry and Steitz, 1987; Pillai et al., 2003), whereas non-coding RNAPII transcripts, such as small nuclear RNAs (snRNAs), are processed by the Integrator complex (Baillat et al., 2005). The Integrator was discovered as a factor required for 3′ end processing of metazoan snRNAs (Baillat et al., 2005), which critically depend on snRNA promoters driving transcription (Hernandez and Weiner, 1986; Lobo and Hernandez, 1989), and the Integrator is believed to couple both processes (Egloff et al., 2007). More recent studies suggest that transcription termination of human snRNA genes may also occur in an Integrator-independent manner (Davidson et al., 2020).

Since its discovery, the Integrator has been shown to be involved in several other pathways, including biogenesis of enhancer RNAs (Lai et al., 2015), telomerase RNA (Rubtsova et al., 2019), long non-coding RNAs (Nojima et al., 2018), viral microRNA (miRNA) biogenesis during *Herpesvirus saimiri* infection (Cazalla et al., 2011; Xie et al., 2015), as well as regulation of the RNAPII transcription pause/release cycle (Beckedorff et al., 2020; Gardini et al., 2014; Rienzo and Casamassimi, 2016; Skaar et al., 2015; Yamamoto et al., 2014). Most recent studies show that the Integrator can destabilize promoter-proximally paused RNAPII, leading to nascent RNA cleavage and transcription attenuation in a wide range of protein-coding genes (Elrod et al., 2019; Lykke-Andersen et al., 2020; Tatomer et al., 2019).

Given such a broad spectrum of functions, it is not surprising that several genetic disorders have been associated with mutations in the components of the Integrator complex (Krall et al., 2019; Oegema et al., 2017; Zhang et al., 2020a).

To date, 14 unique proteins have been identified as core components of the Integrator complex (Baillat et al., 2005; Chen et al., 2012), some of which interact with additional factors (Barbieri et al., 2018; Huang et al., 2020). None of the core Integrator subunits (INTS1–INTS14) are shared with the CPSF or the histone pre-mRNA processing machinery (Baillat and Wagner, 2015). However, based on sequence similarities, INTS9 and INTS11 have been identified as homologs of CPSF100 and CPSF73, respectively (Dominski et al., 2005b). Both proteins belong to the family of metallo-β-lactamase (MBL)/β-CASP (CPSF-Artemis-SNM1-Pso2) nucleases, which play important roles in various aspects of the RNA metabolism (Pettinati et al., 2016). Nucleases from this family are characterized by the presence of seven conserved sequence motifs (1–4 in the MBL and A–C in the β-CASP domains) (Dominski et al., 2013) typically coordinating two catalytic Zn^{2+} ions within the active site, located in a deep cleft between the MBL and β-CASP domains

This is an open access article under the CC BY license (http://creativecommons.org/licenses/by/4.0/).

(Mandel et al., 2006). Those sequence motifs, including the characteristic HxHxDH consensus sequence (motif 2), are conserved in INTS11 but not in INTS9, implying that the two proteins form a pair of active (INTS11) and catalytically impaired (INTS9) nucleases, reminiscent of CPSF73 and CPSF100 (Dominski et al., 2005b; Mandel et al., 2006). Indeed, mutations in the putative active site of INTS11 result in snRNA misprocessing and identify it as the *bona fide* catalytic subunit of the Integrator complex (Baillat et al., 2005).

Yeast two-hybrid and immunoprecipitation experiments showed that INTS9 and INTS11 form a heterodimer and do not cross-react with CPSF73 or CPSF100 (Albrecht and Wagner, 2012; Dominski et al., 2005b). The interaction of INTS9 and INTS11 is mediated by the C-terminal regions of both proteins and is necessary for proper pre-snRNA processing and stable association with other integrator subunits (INTSs) (Albrecht and Wagner, 2012; Wu et al., 2017). INTS4, a HEAT repeat-containing protein, has been identified as such a direct interaction partner of the INTS9/11 dimer (Albrecht et al., 2018). RNAi-mediated depletion of all three proteins results in a higher degree of pre-snRNA misprocessing than for any other INTS, suggesting that they form a minimal INTS4/9/11 core complex, hereafter referred to as the Integrator cleavage module (Albrecht et al., 2018). In the analogous mammalian cleavage factor (mCF) and in the histone cleavage complex (HCC), the CPSF73/100 dimer interacts directly with the HEAT repeat protein Symplekin, forming a catalytic core shared between the two machineries (Kolev and Steitz, 2005; Michalski and Steiniger, 2015; Sullivan et al., 2009). This, together with similarities in the primary sequence motifs, suggests that INTS4 and Symplekin might be functionally related factors (Albrecht et al., 2018).

In the past few years, tremendous progress has been made in structural studies of the 3′ end-processing machineries, providing first insights into the architecture of the substrate recognition modules of the cleavage and polyadenylation factor (CPF)/CPSF (Casañal et al., 2017; Clerici et al., 2018; Sun et al., 2018), their recruitment to the mCF (Zhang et al., 2020b) and activation of CPSF73 within a fully assembled histone pre-mRNA processing complex (Sun et al., 2020).

In contrast, very little is known about the molecular architecture of the Integrator complex despite its emerging importance for transcription attenuation and 3′ end processing of a wide range of substrates. The structure of a small INTS9/11 C-terminal domain 2 (CTD2) dimer provided first detailed insights into one of several interfaces between these two proteins (Wu et al., 2017); however, the majority of the cleavage module remains structurally uncharacterized. This poses several questions regarding the relative orientation of the two nuclease domains, the role of the INTS4 in assembly of the cleavage module, the mechanism of substrate recognition and nuclease activation, and how the specificity is achieved.

Here we analyzed the composition of the native Integrator complex, identified several stable Integrator sub-complexes, and report a 3.5-Å cryoelectron microscopy (cryo-EM) reconstruction of the 250-kDa INTS4/9/11 ternary complex. Our structure provides insights into the molecular architecture of the Integrator cleavage module, revealing a tight association of INTS9 and INTS11 and a stabilizing role of INTS4 for several mobile domains. Comparison with other 3′ end-processing machineries highlights the unique architecture of the Integrator's catalytic core.

RESULTS

Modularity of the Integrator complex

The Integrator complex consists of 14 different subunits (Baillat et al., 2005; Chen et al., 2012), but little is known about its internal architecture or the assembly mechanism. To gain more insights into the composition of the Integrator complex, we generated a series of stable HEK293F cell lines ectopically overexpressing tagged variants of INTS4, INTS5, INTS7, INTS10, and INTS14 (data for INTS7 and INTS14 not shown). We performed tandem affinity purification followed by quantitative mass spectrometry analysis to identify factors co-purifying with each bait protein (Figure 1A). INTS4 pull-down was used as an internal reference against which all enrichment ratios were calculated.

Using a stringent tandem affinity purification approach, we observed that each bait protein enriched a different subset of INTSs. INTS8 co-purified mainly with INTS5, whereas INTS10 co-purified INTS13 and INTS14. Similarly, INTS4 showed co-enrichment with INTS9 and INTS11 compared with two other pull-downs (Figures 1B–1E). Other INTSs were also detected in our experiment, but their enrichments were significantly lower.

Because INTS4/9/11 has been described previously as a stable module of the Integrator (Albrecht et al., 2018), we concluded that two other assemblies detected here, INTS5/8 and INTS10/13/14, may also be similar, independent modules. To validate the newly identified subcomplexes, we expressed them in insect cells and purified and analyzed them by size-exclusion chromatography (SEC) (Figures 1F–1I). Each of the newly identified complexes eluted as a single symmetrical peak (Figures 1G and 1I), indicating its biochemical homogeneity.

To reconstitute a larger core complex, we mixed all three modules at an equimolar ratio and subjected them to SEC. We observed a slight shift in the elution volume, which was also observed when mixing only INTS4/9/11 and INTS10/13/14, indicating that INTS4/9/11 and INTS10/13/14 interact with each other but not with INTS5/8 (Figures S1B–S1D). This interaction was confirmed further when the modules INTS4/9/11 and INTS10/13/14 were co-expressed in Hi5 cells and purified by tandem affinity purification with one tag on each module (Figure S1A)

Overall architecture of the Integrator cleavage module

A ternary complex of INTS4/9/11 was produced by co-expression of full-length proteins in insect cells, followed by tandem affinity purification with an 8×His tag attached to INTS4 and streptavidin-binding peptide (SBP) tag to INTS11 (Figures S2A and S2B). Using single-particle cryo-EM, we obtained a 3D reconstruction of the complex at an overall resolution of 3.5 Å (Figures 2 and S3–S6; Table 1).

The body of the structure is composed of two MBL/β-CASP domains of INTS9 (residues 1–506) and INTS11 (residues 1–449) facing each other in a head-to-head arrangement with a pseudo 2-fold symmetry axis running along their interface (Figures 2A and 2B). The C termini of INTS9$^{507–556}$ and INTS11$^{450–502}$ extend from the body of the complex and form a tightly intertwined composite domain, hereafter referred to as the CTD1 dimer. A small

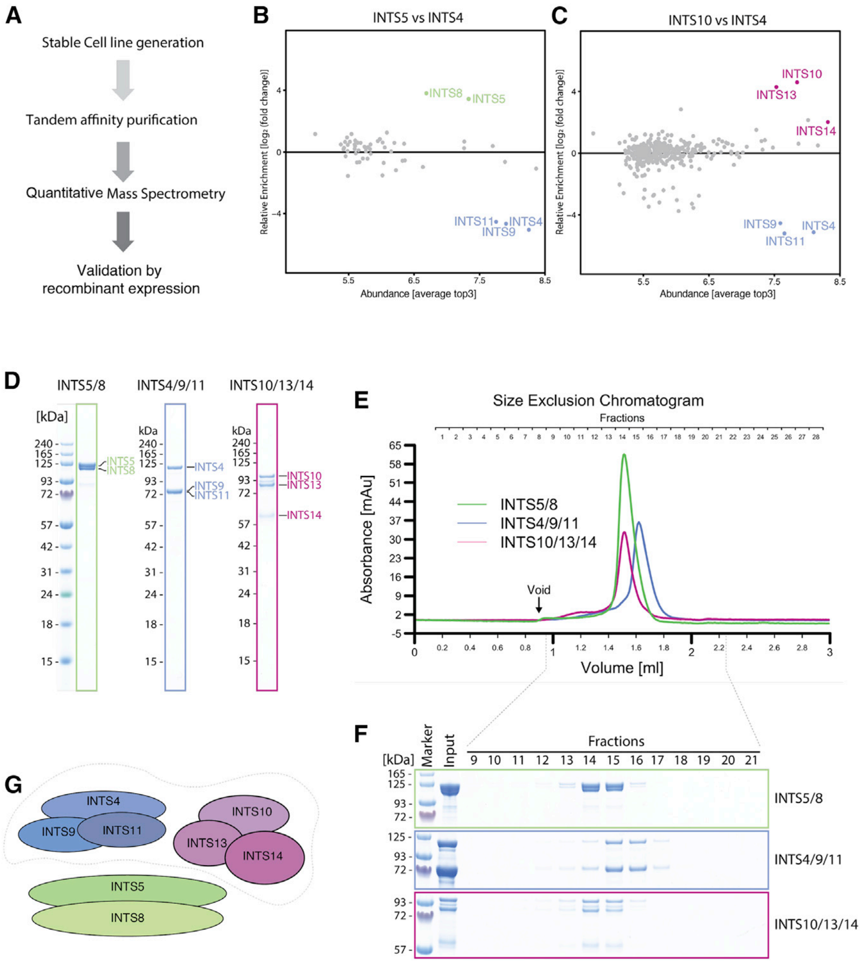

Figure 1. Modularity of the Integrator complex

(A) Schematics of the workflow used for identification of new Integrator sub-complexes.

(B and C) Quantitative mass spectrometry experiments showing co-enrichment of the potential interaction partners by each bait protein. The relative enrichment (compared with the INTS4 pull-down reference) is plotted against the average Top3 value, which estimates the abundance of each protein. A complete list of hits is available in Table S1.

(D) SDS-PAGE of the recombinant Integrator modules co-expressed and purified from insect cells.

(E) SEC of INTS5/8, INTS4/9/11, and INTS10/13/14.

(F) Fractions of the SEC shown in (E), corresponding to the absorbance peak as indicated.

(G) A schematic of the identified modules. The dotted line around modules INTS4/9/11 and INTS10/13/14 refers to the interactions shown in Figure S1.

linker helix at the C-terminus of INTS9^{CTD1} (residues 496–502) is also present in a recently reported structure of the INTS9/11 C-terminal regions (INTS9$^{582–658}$/INTS11$^{503–600}$), which we define as the CTD2 dimer (Wu et al., 2017). Superposition of the two structures, together with focused classification and refinement, allowed unambiguous placement of the CTD2 dimer within our cryo-EM reconstruction (Figures 2C, 2D, and S5A).

An α-helical repeat that wedges between the two nuclease domains has been identified as the N-terminal domain of INTS4 (INTS4^{NTD}), consistent with the visible side-chain densities, secondary structure predictions, and cross-linking and mass spectrometry data (Figures 2 and 4). INTS4^{CTD} is not well resolved in our map, but the density near the INTS9$^{\beta\text{-CASP}}$ domain could be unambiguously assigned to this part of the protein.

INTS9 and INTS11 form a tight dimer via multiple interfaces

INTS9 and INTS11 share 21.7% sequence identity and a similar domain architecture. In both proteins, a MBL/β-CASP nuclease domain constitutes the largest part of the protein and is followed by the smaller CTD1 and CTD2. Heterodimerization of INTS9 and INTS11 is achieved through several spatially distributed contacts involving each of the three domains, together accounting for 2,944 Å^2 of buried surface area (BSA) (Figure 3). In the resulting pseudo-symmetric arrangement, CTDs wrap around one another and bring the nuclease domains into close proximity. Despite facing each other at a close distance, the nuclease domains of INTS9 and INTS11 (107 kDa combined) form only minor contacts (400 Å^2 BSA) and do not contribute significantly to heterodimer formation (Figures 3A–3C and S1H).

INTS9^{CTD1} and INTS11^{CTD1}, in contrast, form a composite domain and account for a significantly larger interaction surface despite its small size (1,200 Å^2 BSA, 11.5 kDa). The structure of the CTD1 dimer resembles a β-barrel with hydrophobic residues facing its inner core (Figures 3D and 3E). Extensive interactions between these two domains are achieved via formation of two intermolecular β-sheets involving INTS9$^{CTD1\text{-}\beta1}$ and INTS11$^{CTD1\text{-}\beta1}$ as well as INTS9$^{CTD1\text{-}\beta32}$ and INTS11$^{CTD1\text{-}\beta2}$, which connect the two halves of the barrel (Figure 3E). Formation of the CTD1 dimer tethers the two nuclease domains together and restricts their movement, facilitating formation of other weak interactions.

Another major contact involves INTS9^{CTD2} and INTS11^{CTD2}, which form the second independent dimerization domain, as reported previously (Wu et al., 2017). The CTD2 region appears flexible in our cryo-EM reconstruction, and it was modeled entirely by rigid body docking of the available crystal structure.

Formation of the INTS9/11 CTD1 dimer is functionally important and necessary for assembly of the cleavage module

To analyze to what extent CTD1 dimer formation is important for Integrator function, we generated a series of INTS11 variants that aimed to disrupt the interface observed in the structure. We screened those variants for the effect on RNA processing *in vivo*

CellPress
OPEN ACCESS

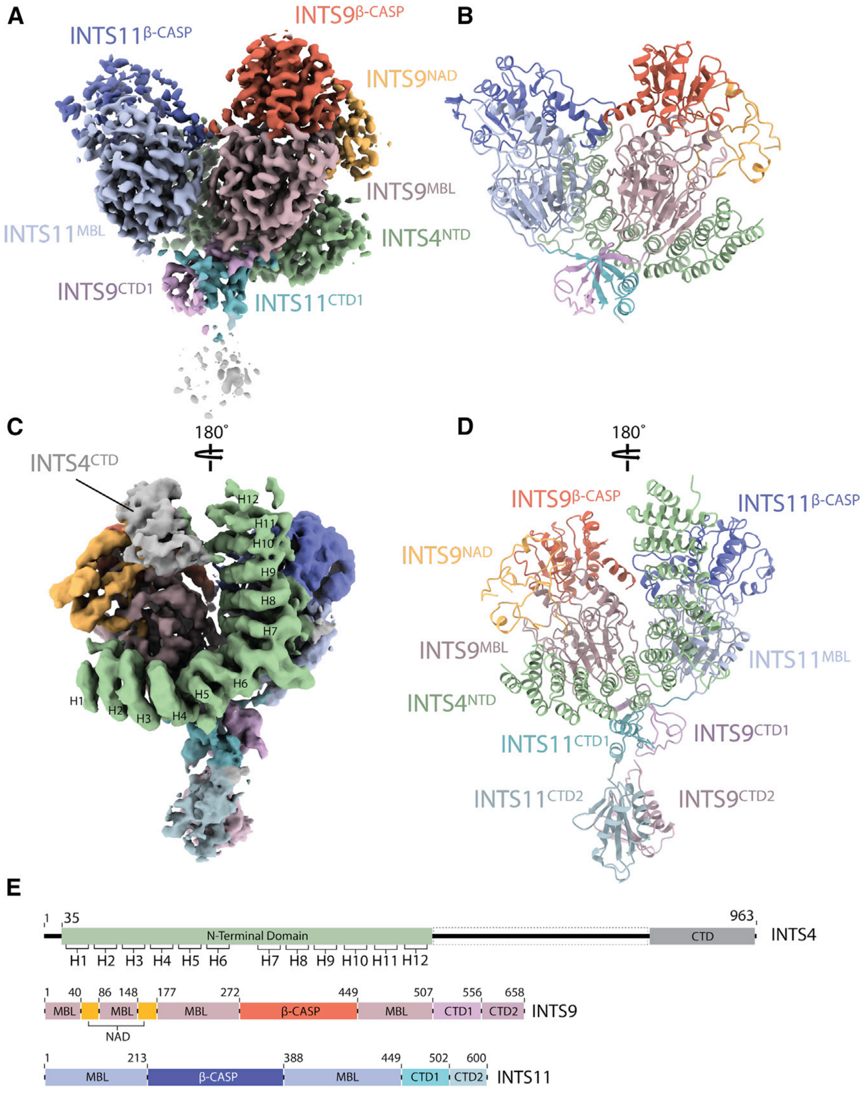

Figure 2. An overview of the INTS4/9/11 structure

(A) Experimental cryo-EM density of the high-resolution map, colored according to subunits and domain identity. INTS11 is shown in blue (β-CASP, dark blue; MBL, light blue; CTD1, cyan), INTS9 in red (β-CASP, dark red; MBL, light red; CTD1, pink; INTS9^{NAD}, yellow), and INTS4^{NTD} in green.

(B) A cartoon representation of the atomic model built into the high-resolution map; shown is the same view as in (A).

(C) Back view of the cryo-EM density obtained by processing the ESRF1 dataset alone, showing additional HEAT repeats in INTS4^{NTD} and an additional density for the INTS4^{CTD} and INTS9/11 CTD2 domain.

(D) A complete model of INTS4^{NTD}/INTS9/INTS11 in the same orientation as in (C).

(E) Sequence bars showing the domain organization of all three proteins.

See Video S1.

in a depletion/reconstitution assay utilizing the U7 snRNA reporter (Albrecht and Wagner, 2012). In this experimental setup, impaired 3′ end cleavage of the U7 transcript results in transcriptional readthrough, allowing translation of the downstream GFP gene, which can be quantified by fluorescence intensity measurement (Figure 3G).

Deletion CTD2 or CTD1 and CTD2 domains of INTS11 has a significant effect on the processing activity of the Integrator (Figure 3H). This is to be expected, considering that CTD2 is required for dimerization of INTS9/11, as shown previously (Wu et al., 2017). Deletion of INTS11^{CTD1} alone has a severe effect on reporter RNA misprocessing, even though this domain is not required for dimerization of INTS9/11, as analyzed by co-expression and pull-down of the 3×hemagglutinin (HA)-INTS9 and 3×FLAG-INTS11 protein variants (Figure S1F). To analyze this further, we designed a series of point mutations in INTS11^{CTD1} in which pairs of amino acids located at the interface of the intermolecular β-sheet were substituted with prolines. These mutations are predicted to disrupt the secondary structure and, consequently, formation of the composite β-barrel-like domain. Although none of the introduced point mutations affected its dimerization properties (Figure S1F), they increased reporter RNA misprocessing to levels comparable with deletion of the entire INTS11^{CTD1} or introduction of the catalytic mutation E203Q at the INTS11 active site (Figures 3H and 3I). This suggests that CTD1 dimer formation is important for Integrator function.

We hypothesized that this domain might be important for assembly of higher-order complexes, providing a quality control checkpoint. INTS4 recruitment control would be a likely target for such a checkpoint. To test this hypothesis, we co-expressed SBP-INTS4 with 3×HA-INTS9 and 3×FLAG-INTS11 variants in HEK293T cells, followed by HA-agarose pull-down and western blotting. Deletion of the CTD2 or CTD1 and CTD2 regions of INTS11 abolishes binding to INTS4 even though the nuclease domain of INTS11 has a significant interface with INTS4. Deletion of INTS11^{CTD1} has no effect on dimerization of INTS9 and INTS11(Figure 4A) but fails to enrich INTS4 in the pull-down experiment (Figure 4A). Consistently, point mutations, which disrupt CTD1 dimer formation, abolish recruitment of INTS4 (Figure 4A, lanes 7–9). The fact that these residues are not involved in interactions with INTS4 suggests that proper CTD1 dimer formation is required before INTS9/11 can be incorporated into the Integrator complex.

INTS4 acts as a scaffold for the INTS9/11 dimer

Secondary structure predictions show that INTS4 is almost an entirely α-helical protein, with the exception of the very C-terminal 150 residues, which display a high propensity to form β

Table 1. Cryo-EM data collection, model refinement, and validation statistics

	High-resolution EMDB: EMD-12165 (PDB: 7BFP)	Medium-resolution (ESRF1) EMDB:EMD-12166 (PDB: 7BFQ)	INTS4-focused EMDB: EMD-12164 (PDB: 7BFQ)	CTD2-focused EMDB: EMD-12163 (PDB: 7BFQ)
Data collection and processing				
Magnification	165,000	165,000	165,000	165,000
Voltage (kV)	300	300	300	300
Electron exposure (e–/Å^2)	42–46.8	46.8	46.8	46.8
Defocus range (μm)	−0.5 to −3.0	−0.5 to −3.0	−0.5 to −3.0	−0.5 to −3.0
Pixel size (Å)	0.83	0.83	0.83	0.83
Symmetry imposed	C1	C1	C1	C1
Initial particle images (no.)	9,124,445	1,017,432	1,017,432	1,017,432
Final particle images (no.)	26358	21,866	21,235	19,938
Map resolution (Å)	3.	4.1	6.5	6.5
FSC threshold	0.143	0.143	0.143	0.143
Map resolution range (Å)	3.4–5	4–6	5–9	5–9
Refinement				
Model resolution (Å)	4.1 (average)			
FSC threshold	0.5			
$FSC_{average}$(Refmac)	0.7 (at 3.5 Å)			
Model resolution range (Å)	3.4–5			
Map sharpening *B* factor (Å^2)	−50			
Model composition				
Non-hydrogen atoms	10,668			
Protein residues	1340			
B factors (Å^2)				
Protein	148			
RMSDs				
Bond lengths (Å)	0.01			
Bond angles (°)	0.16			
Model validation				
MolProbity score	2.2			
Clashscore	8.7			
Poor rotamers (%)	2.4			
Ramachandran plot				
Favored (%)	93.1			
Allowed (%)	6.5			
Disallowed (%)	0.4			

strands. Of 37 helical segments predicted in INTS4, 24 form helix-turn-helix motifs, which are clearly visible in our maps, and seven of them exhibit sequence signatures of a canonical HEAT repeat (Andrade et al., 2001).

Twelve HEAT repeat motifs (H1–H12) are well defined in our structure and constitute INTS4^{NTD}, which forms a curved solenoid-like structure (Figures 2C–2E). The inner, concave surface of INTS4^{NTD} is in contact with the MBL domain of INTS9, and H1–H5 interact with the INTS9^{MBL} domain by forming specific polar contacts (Figures 4B–4E). Other prominent contacts include the charged residues at the tip of H5, which interact with the CTD1 dimer, tethering it into its position (Figure 4E). Additional contacts with the CTD1 dimer are formed by H6, which also forms a contact with INTS11^{MBL}.

The outer, convex surface of H7–H12 faces the nuclease domain of INTS11, but the quality of the map in this region does not allow us to confidently assign an amino acid register. It is possible that additional contacts between the two proteins exist.

Importantly, the simultaneous interaction of INTS4^{NTD} with INTS9^{MBL} and INTS11^{MBL} via its inner and outer surfaces locks the relative orientation of the two nuclease domains, which would otherwise be associated loosely. Similarly, the CTD1 dimer does not form any contacts with the nuclease domains

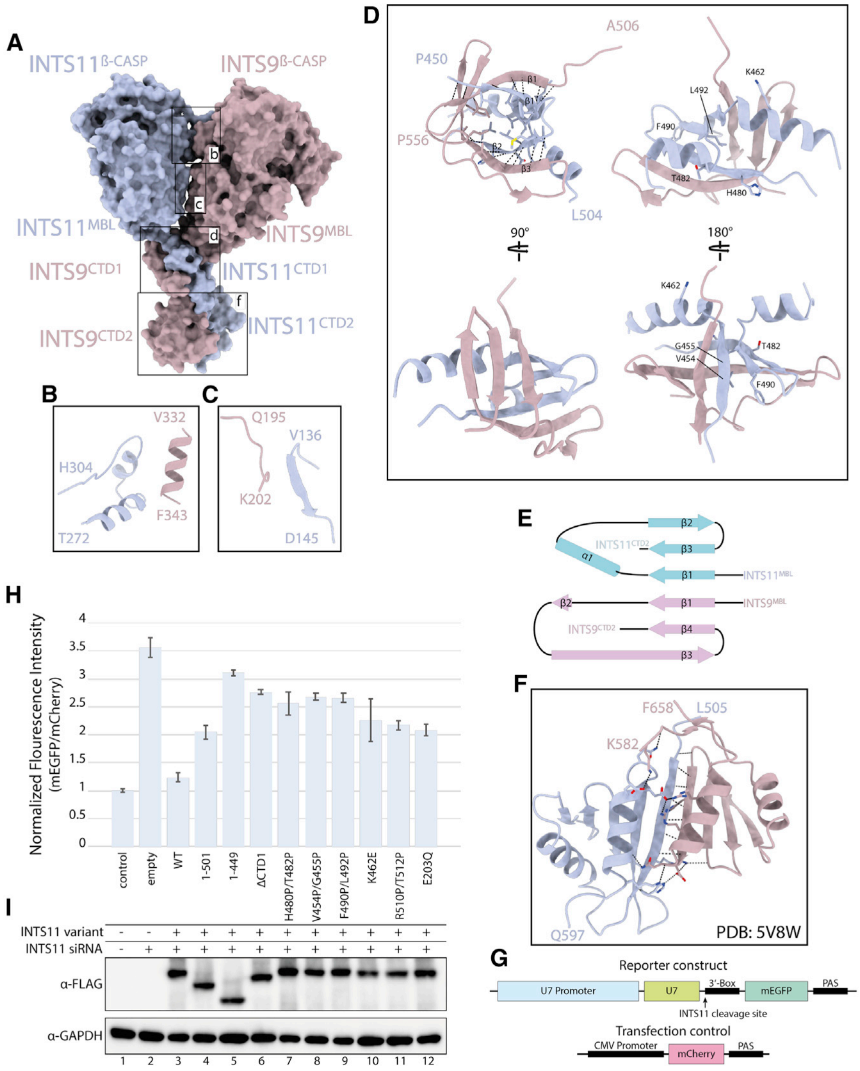

Figure 3. Structural basis of INTS9/11 heterodimerization

(A) Surface representation of the model, showing contact areas. Interfaces involved in dimer formation are highlighted with close ups.

(B and C) Insets showing the proximity of the two nuclease domains; no polar contacts could be identified between these regions.

(D) A β-barrel-like structure formed by the CTD1 domains of INTS9 and INTS11, highlighting intermolecular β-sheets formed by the two proteins.

(E) A topology plot of the CTD1 dimer, showing the intermolecular β-sheet.

(F) CTD2 dimer of INTS9 and INTS11, as reported previously (PDB: 5V8W).

(G) Schematics of the constructs used in the GFP-based U7 snRNA *in vivo* processing assay.

(H) mEGFP/mCherry fluorescence intensity readout from the depletion/rescue experiment assessing the functionality of different INTS11 variants. "Empty" refers to the condition where no protein was overexpressed. Error bars represent standard deviation from 3 individual measurements.

(I) Western blot showing protein expression levels of the transgenes used in the depletion/rescue experiment (H).

and remains largely independent. By interacting with a loop in the INTS9^{CTD1} β-sheet, INTS4^{NTD} tethers the entire CTD1 dimer toward INTS9, stabilizing the entire assembly in a well-defined configuration (Figures 4E).

INTS4^{CTD} could not be traced directly from the end of the INTS4^{NTD}; however, our medium-resolution map (Figures 2C, S6E and S6F) shows an additional density adjacent to the INTS9$^{β\text{-}CASP}$ domain, which could only be interpreted as the missing INTS4^{CTD}. Although we could not interpret this density with an atomic model, its location reveals that INTS4 interacts with the INTS9/11 heterodimer using two independent domains linked with a flexible region (Figures 2). Such bimodal binding is supported by previous yeast two-hybrid studies (Albrecht et al., 2018) and could have important consequences for complex assembly and substrate recruitment.

The INTS11$^{MBL/β\text{-}CASP}$ domain exhibits features of an inactive conformation

The INTS11^{MBL} domain is composed of a typical αββα-fold, with the β-CASP domain inserted into one of its loops (Figure S7). The nuclease domain of INTS11 shares 40% sequence identity with CPSF73, and the invariant residues include all 7 sequence motifs (1–4 in the MBL and A–C in the β-CASP domains (Figure S7), which, in CPSF73, coordinate two catalytic Zn^{2+} ions within a deep cleft between the MBL and β-CASP domains (Mandel et al., 2006). The geometry of these motifs is preserved in INTS11 and capable of supporting a similar coordination of the catalytic Zn^{2+} ions (Figures 5D and 5E). This implies that INTS11 forms a *bona fide* MBL/β-CASP nuclease active site.

The relative orientation of the MBL and β-CASP domains of INTS11 resembles that observed for CPSF73 (Mandel et al., 2006) and its homologs Ysh1 (Hill et al., 2019) and CPSF3 (Swale et al., 2019). In this configuration, a cleft leading to the active site is too narrow to accommodate the RNA substrate, suggesting that the nuclease was captured in a closed, inactive conformation (Figure 5). The local resolution for the INTS11$^{β\text{-}CASP}$ domain is relatively low (Figure S6), indicating the dynamic nature of this region. It is consistent with the idea that displacement of the β-CASP domain would allow it to achieve an active configuration similar to the one observed for CPSF73 (Sun et al., 2020) or RNase J (Dorléans et al., 2011).

The INTS9 $^{MBL/β\text{-}CASP}$ domain contains non-canonical insertions

INTS9 exhibits a domain architecture similar to INTS11; however, most of its conserved sequence motifs responsible for creating the active site are mutated (Figures 5C and S7). In

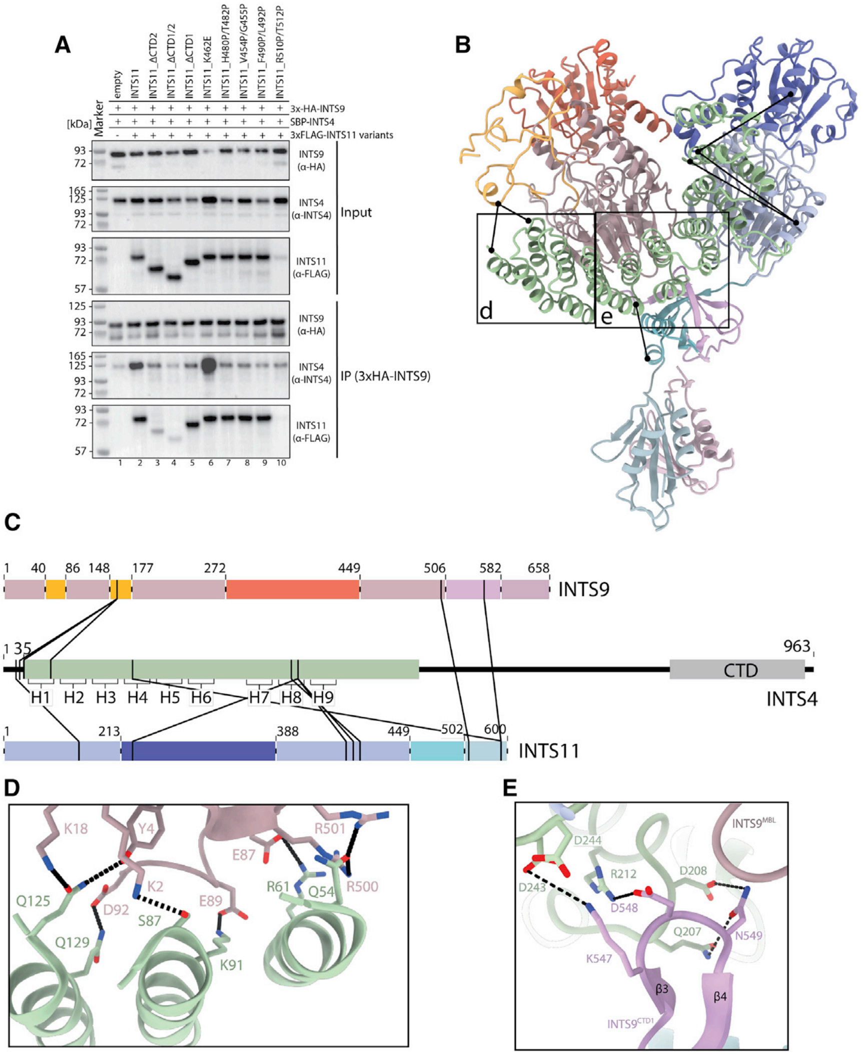

Figure 4. INTS4 recruitment to the cleavage module and its scaffolding role in organizing mobile domains of the complex

(A) Western blot showing the results of the HA-agarose pull-down experiments from HEK293T cells co-expressing the 3×HA-INTS9, SBP-INTS4, and 3×FLAG-INTS11 variants. Deletion of CTD2 or CTD1/2 (lanes 3 and 4) as well as point mutations in CTD1 (lanes 7–9) result in reduced recruitment of INTS4 compared with wild-type (WT) INTS11 (lane 2). INTS11^{K462E} (lane 6) was not meant to disrupt CTD1 and recruitment of the INTS4. Elevated levels of INTS4 in this pull-down likely originate in higher INTS4 levels already in the input sample and/or additional electrostatic interactions between newly introduced 462E and the neighboring positively charged surface of INTS4.

(B) Model of the cleavage module, showing intermolecular cross-links with a distance of less than 35 Å.

(C) Cross-linking and mass spectrometry (XL-MS) data mapped along the sequence of each protein.

(D and E) Interactions of the INTS4^{NTD} with the nuclease heterodimer.

particular, the HxHxDH sequence in catalytic motif 2 is changed to NYHC, not only eliminating 3 of 4 ligands for the catalytic Zn^{2+} ions but also causing significant changes in the geometry of the active site. This and other alterations render the INTS9$^{MBL/\beta\text{-}CASP}$ domain incapable of coordinating catalytic divalent ions, reinforcing the notion that INTS9 is a pseudoenzyme.

INTS9 and CPSF100 share 23% sequence identity, and their superposition reveals a very good correspondence of their tertiary structures (root-mean-square deviation [RMSD] = 1.8 Å). Despite this similarity, INTS9 has some unique features that are not present in other MBL/β-CASP proteins. The most prominent are two large loops inserted into the canonical MBL fold, with the first one (residues 40–82) between helix 4 and β strand 5 and the second (residues 148–192) between helices 2 and 3 (Figures 2 and 7). These two loops are well-ordered in our structure, interact with each other, and together form a structurally distinct domain in INTS9, referred to as the INTS9 accessory domain (INTS9^{NAD}). Although conserved among Integrator sequences, the functional implications of these insertions remain unknown.

Structural implications for RNA substrate binding

We performed electrophoretic mobility shift assays (EMSAs) to assess the RNA-binding properties of INTS4/9/11 and other reconstituted sub-complexes (Figures 6A–6C). Our data show that INTS4/9/11 can form an RNP complex with its cognate pre-U1 snRNA substrate, containing the 3′-box signal sequence (Ciliberto et al., 1986; Hernandez, 1985; Yuo et al., 1985), but also with an unstructured single-stranded RNA (ssRNA) (Figure 6A). Similarly, INTS10/13/14 and INTS5/8 (to a lesser extent) exhibit RNA binding properties (Figures 6B and 6C). However, in all three cases, the binding appears to be non-specific and in a low-affinity range. The lack of sequence specificity in RNA binding is consistent with the genome-wide functions of the Integrator and a broad spectrum of different substrates.

Next we wanted to find out whether INTS4^{NTD} could be involved in substrate binding in the Integrator complex in a manner similar to Symplekin in the HCC. In the histone processing machinery, Symplekin^{NTD} is critical for its activity *in vitro*, and it bridges the CPSF73/100 dimer to the U7snRNP, forming a cavity that accommodates the U7 snRNA:histone pre-mRNA duplex (Sun et al., 2020). Analysis of the surface electrostatic potential of the INTS4/9/11 complex reveals a highly positively charged cavity formed by all three proteins (Figures 6F and 6H). This composite cavity is formed between the INTS9/11 MBL domains and traces along the concave side of INTS4 leading toward the INTS11$^{\beta\text{-}CASP}$ domain (i.e., the nuclease active site). In contrast, a similar charged surface is not present at the equivalent position in the CPSF73/100

CellPress
OPEN ACCESS

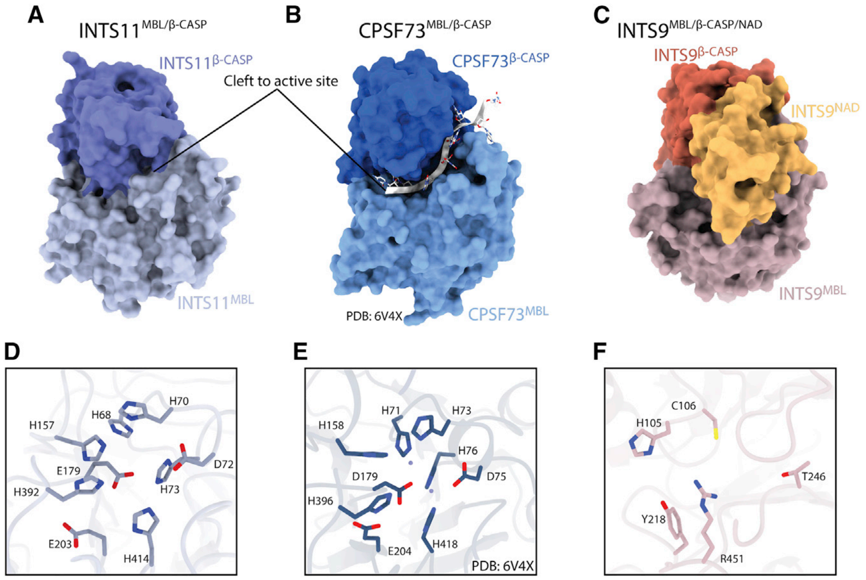

Figure 5. Structure of MBL/β-CASP of INTS9, INTS11, and their homolog CPSF73

(A–C) Surface representation of the 3 nuclease domains, highlighting the cleft leading to the active site of CPSF73 and its absence in INTS11 because of a closed conformation.

(D) Nuclease active site of INTS11, showing that all residues required for coordination of catalytic zinc ions are present.

(E) The active site of CPSF73 (PDB: 6V4X) with the two catalytic zinc ions, shown as spheres.

(F) Disintegrated active site of INTS9, showing the altered geometry and lack of the key metal-binding residues.

complex, and neither do we see any other charged surfaces in the INTS9/11 dimer in a place that would correspond to histone the pre-mRNA binding site in the HCC. Interestingly, the side chains in the helix-loop-helix motifs of INTS4^{NTD} point toward the putative RNA-binding groove in a manner resembling the Symplekin^{NTD}, where the corresponding loops bind the phosphate backbone of the H2a pre-mRNA.

We performed mutagenesis of the charged residues in INTS4 and INTS11, which are involved in formation of this positively charged surface, and analyzed their effect using our U7 snRNA reporter *in vivo*. Of seven point mutations tested, three impair the Integrator's 3′-processing activity (INTS11^{K462E}, INTS4$^{H164A/R167A}$, and INTS4^{R210A}) (Figure 6D). INTS11^{K462E} and INTS4$^{H164A/R167A}$ are located around a highly positively charged composite tunnel formed by INTS9, INTS11, and INTS4 (Figure 6H). INTS4^{R210A} is located in a deep cleft, in close proximity to CTD1 (Figure 6F). The remaining point mutations tested (INTS4^{K217E}, INTS4^{K304E}, and INTS4^{R333E}) are located at the center of the INTS4 HEAT repeat, farther away from the charged tunnel, suggesting that the charge of the groove is less important for RNA processing.

Comparison with the histone pre-mRNA processing machinery reveals unique architectural features of the Integrator complex

The arrangement of the INTS9/11 heterodimer closely resembles the one observed in the related CPSF73/100 complex (Sun et al., 2020), reported to be part of the histone pre-mRNA processing machinery (Figures 7A and 7B). In both complexes, the two respective nucleases are brought together by dimerization of their two consecutive CTDs. Despite a similar overall conformation, the INTS11$^{MBL/β\text{-}CASP}$ domain remains in a closed inactive configuration (Figure 5).

The most striking differences between the two structures are the positions of the HEAT-repeat proteins INTS4 and Symplekin, which are believed to be functionally related (Albrecht et al., 2018). In both cases, they interact predominantly via their N termini with the pseudonuclease (INTS9 or CPSF100) but are located on opposite sides of their respective nuclease heterodimers (Figures 7F–7H). In addition, Symplekin^{NTD} binds head on with only two helices to the β-CASP domain of CPSF100, whereas INTS4^{NTD} stretches along the entire MBL domain of INTS9.

Because of limited resolution, we could not model INTS4^{CTD}, but our map shows a large elongated density associating with the INTS9$^{β\text{-}CASP}$ domain, which we interpreted as the missing INTS4^{CTD} (Figure 2C). The CTD of Symplekin, on the other hand, associates with a distantly located CTD2 dimer of the CPSF73/100 (Sun et al., 2020; Zhang et al., 2020b). The NTDs and CTDs of Symplekin and INTS4 are not connected by a continuous density, indicating that a flexible central region might be functionally important in both cases. Our structure highlights that, despite similar primary sequence architecture and a bimodal binding manner, both proteins are recruited to distinct regions of their respective nuclease heterodimers.

DISCUSSION

The protein composition of the Integrator complex was established using affinity purification followed by western blotting and mass spectrometry (Baillat et al., 2005). Although this approach successfully uncovered factors involved in complex formation, it could not determine its stoichiometry or inter-subunit contacts, and such architectural information remained missing. Other biochemical approaches revealed the association of INTS4/9/11 (Albrecht et al., 2018) as well as INTS3/6 (Jia et al., 2020; Skaar et al., 2009; Zhang et al., 2013). Building on these findings, we developed a method that allowed us to identify two previously unknown sub-complexes, INTS5/8 and INTS10/13/14. The latter was recently confirmed by another study (Sabath et al., 2020), which revealed the structure of the INTS13/14 dimer and its RNA binding properties.

Our findings suggests that the Integrator complex is highly modular, not unlike the related CPSF/CPF complex (Casañal et al., 2017; Zhang et al., 2020b). Furthermore, we provide

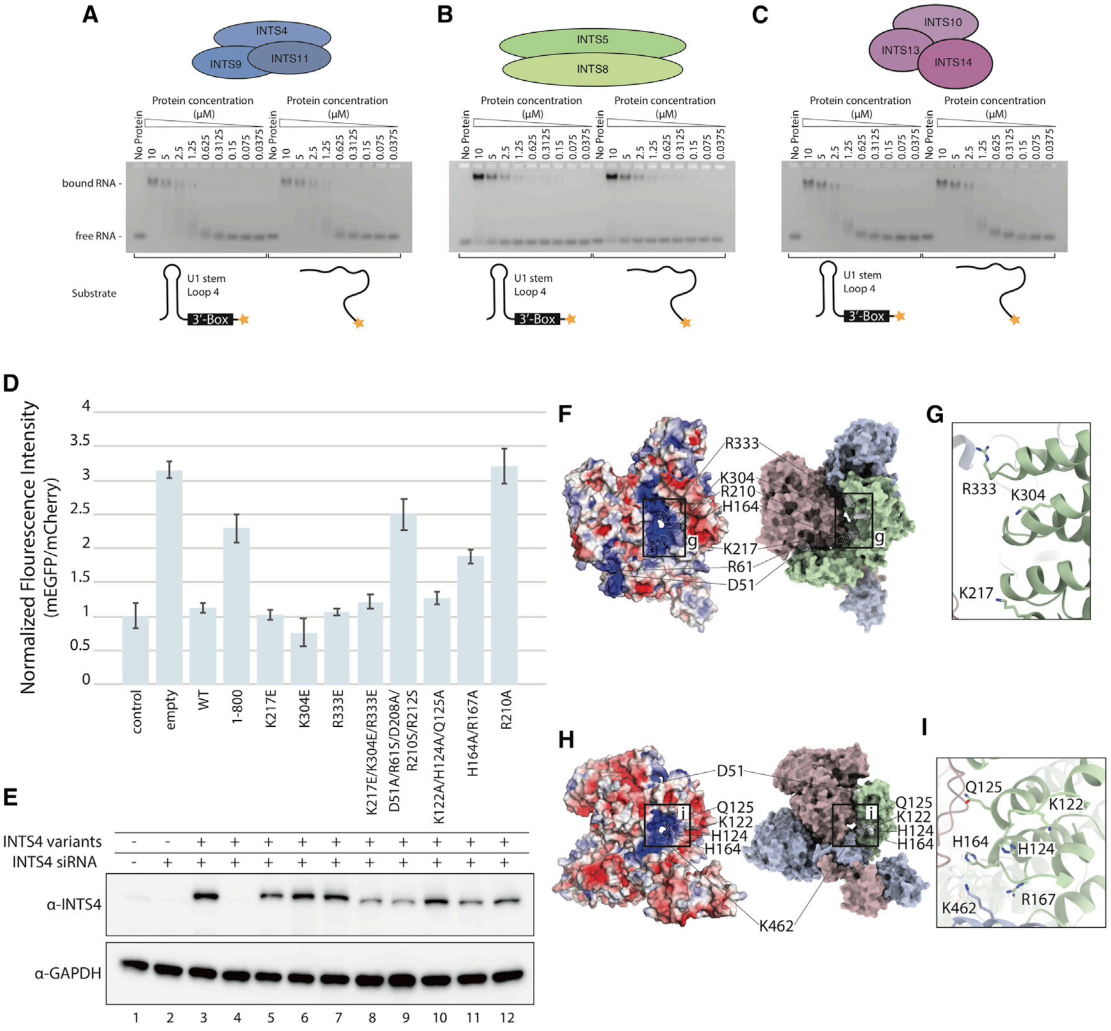

Figure 6. Electropositive surface of INTS4/9/11 and RNA-binding properties of different Integrator sub-complexes

(A–C) EMSA titration assay showing the weak RNA-binding properties of the INTS4/9/11 complex toward a 3′-box-containing U1 pre-snRNA substrate (left panel) and scrambled RNA sequence of the same length (right panel). The same EMSA assay was performed for INTS5/8 (B) and INTS10/13/14 (C).

(D) mEGFP/mCherry fluorescence intensity readout from the depletion/reconstitution experiment assessing functionality of INTS4 variants designed to disrupt the putative RNA-binding surface. "Empty" refers to the condition where no protein was overexpressed. Error bars represent standard deviation from 3 individual measurements.

(E) Western blot showing protein expression levels of the INTS4 variants used in the depletion/reconstitution experiment (D). The lack of signal for INTS4 in lane 4 was expected because the antibody used for detection was raised against the peptide comprising residues 913–963.

(F and H) Surface electrostatic potential calculated with Adaptive Poisson-Boltzmann Solver (APBS) of the INTS4/9/11 complex, showing the location of the point mutations tested with the reporter system.

(G and I) Magnified view of the structural environment of the discussed residues.

evidence that two of the modules (INTS4/9/11 and INTS10/13/14) interact with each other (Figure S1), consistent with recent reports (Mascibroda et al., 2020; Sabath et al., 2020). Notably, other INTSs were also detected in our experiment, but their abundance was much lower compared with the three complexes discussed. It is possible that those subunits are required to bridge the three modules and act as the limiting factors for the complex assembly. INTS7 would be a prime candidate for such a subunit because it is highly abundant in our MS experiment but equally enriched by INTS5/8 and INTS4/9/11 (Figures 1B and 1C).

The association of the Integrator complex with 3′ end processing was initially based on the similarity between INTS9/11 and a nuclease dimer of CPSF73/100 found in the HCC and CPSF (Dominski et al., 2005a). Our structure confirms that each dimer consists of four independent elements: a pseudonuclease domain, a *bona fide* MBL/β-CASP domain, and two separate CTD regions (CTD1 and CTD2) (Figures 7A and 7B). In both cases, dimerization is driven by their tightly entangled CTDs, which bring the nuclease domains into close proximity.

From multiple INTS9-INTS11 interfaces, disruption of the CTD2 dimer has been shown to abolish binding of both

CellPress
OPEN ACCESS

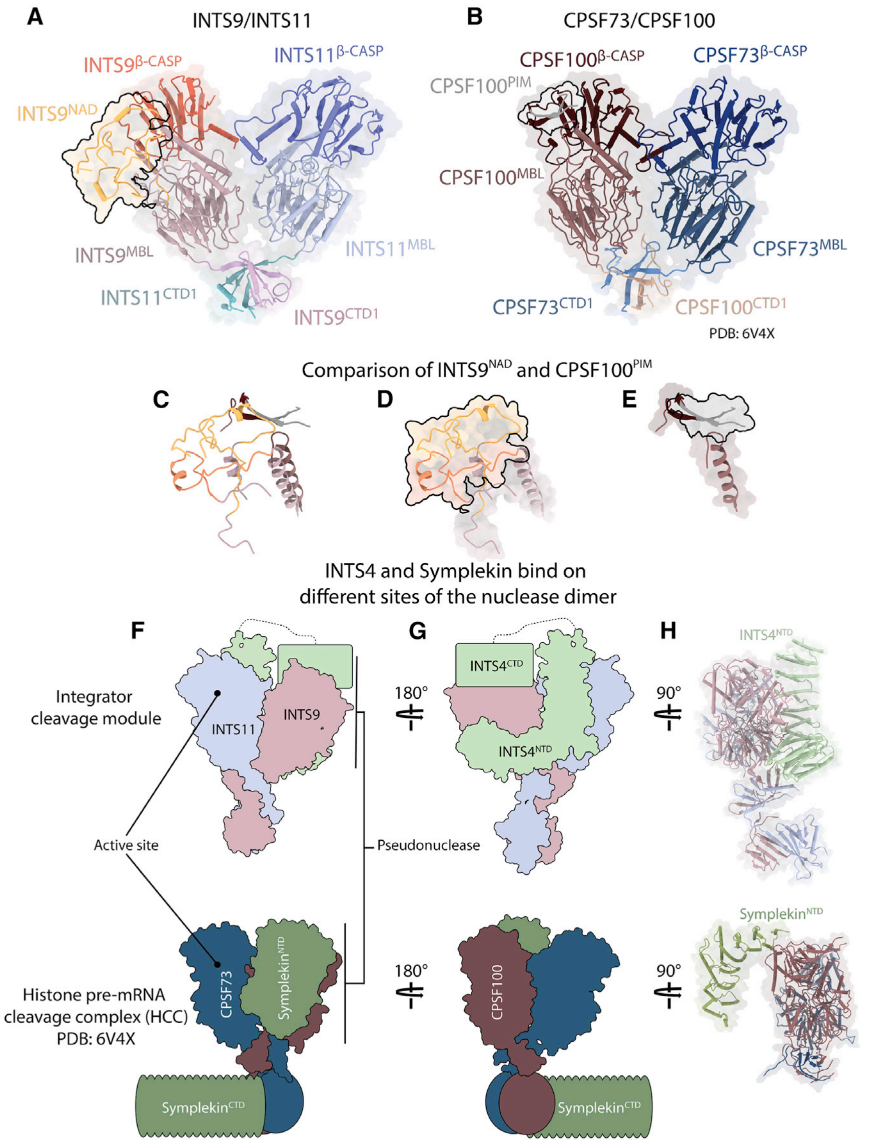

Figure 7. The Integrator cleavage module and HCC

(A and B) Front view of the structure of the nuclease heterodimers (INTS9/11 and CPSF73/100), showing overall good agreement between the two structures and similar positions of the INTS9^{NAD} and CPSF100^{PIM} domains (PDB: 6V4X).

(C–E) Close up and superposition of the CPSF100- and INTS9-specific inserts. The two independent chains of INTS9^{NAD} are colored yellow (40–86) and orange (148–177).

(F) Schematic depiction of the differences in positioning of INTS4 and Symplekin in their respective complexes.

(G) Side view of the INTS4/9/11 complex.

(H) Side view of the CPSF73/100/Symplekin complex, shown in the same orientation as INTS4/INTS9/INTS11 in (D), highlighting different positions of INTS4^{NTD} and Symplekin^{NTD}.

proteins *in vivo* and, consequently, recruitment of other INTSs (Wu et al., 2017). This implies that an intact CTD1 is not sufficient for maintaining the interaction in the absence of the CTD2 dimer interaction or that the CTD1 dimer interface is not formed in this context.

Given the extensive CTD1 dimer interface, we believe the latter is true and that formation of the CTD2 dimer is a prerequisite for establishing the secondary CTD1 dimer interface. Indeed, impaired formation of CTD1 does not affect the interaction between INTS9 and INTS11, but it has severe consequences for recruitment of INTS4, implying that its formation plays a role in assembly of a higher-order complex. It is tempting to speculate that assembly of the INTS9/11 heterodimer may involve progressive formation of multiple interfaces nucleating with the CTD2 dimer, followed by the CTD1 dimer, which brings together two nuclease domains and allows them to form weak interdomain contacts.

Structures of MBL/β-CASP nucleases reported to date show that the β-CASP domain acts as a lid that can adopt an open (Dorléans et al., 2011; Sun et al., 2020) or closed (Hill et al., 2019; Mandel et al., 2006) conformation, enabling or preventing access of the substrate to the active center. INTS11 falls into the second category, consistent with not being engaged with an RNA substrate (Figure 5A). It has been noted previously that CPSF73 and its yeast homolog Ysh1 require repositioning of the β-CASP domain to achieve catalytic competence (Hill et al., 2019; Mandel et al., 2006). The mechanism of such a rearrangement has been shown recently for the histone pre-mRNA processing machinery (Sun et al., 2020). In this case, Lsm10, a component of the substrate recognizing the U7 snRNP module, provides a loop that wedges between the MBL and β-CASP domains of CPSF73, allowing the active center to be engaged with its cognate substrate (Sun et al., 2020). It is plausible that a similar mechanism might exist for the Integrator complex, but the factor that would trigger such activation remains elusive.

Our work identified a structured insertion in INTS9^{MBL}, referred to as the NAD domain (Figures 2 and 7). To our knowledge, this domain is unique to INTS9 and is not present in any other member of the MBL/β-CASP family. Notably, CPSF100 contains an unrelated, disordered insertion (approximately 145 residues) in a different region of the β -CASP domain (Figures 7A–7E and S7). This insertion has been shown recently to contain a linear sequence motif (mPSF interaction motif [PIM]) that is crucial for recruitment of the mammalian polyadenylation specificity factor to the mCF (Zhang et al., 2020b). It is tempting to speculate that the NAD in INTS9 might serve a similar purpose for recruitment of other INTSs. If true, then this observation would support a more

general mechanism shared between different 3′ end-processing machineries, where the inactive nuclease subunit mediates interactions with other modules to bring them into proximity of the endonuclease active site.

In a recent report (Zhang et al., 2020b), the nuclease domains of CPSF73 and CPSF100 in the mCF were captured at a wide angle, very different from our arrangement. A similar open arrangement has not been observed for the Integrator complex, but it is possible that such a configuration exists in the absence of INTS4. Our data suggest that INTS4 plays a role in achieving a compact nuclease/pseudonuclease configuration resembling that observed for CPSF73/100 in the histone-processing machinery (Sun et al., 2020).

INTS4 has been identified previously as a “Symplekin-like” factor (Albrecht et al., 2018) because both proteins interact directly with their respective nuclease heterodimers, and the resulting core complexes are critical for the activities of the corresponding machineries (Kolev and Steitz, 2005; Michalski and Steiniger, 2015; Sullivan et al., 2009). INTS4 and Symplekin are mostly α-helical HEAT repeat proteins and appear to be composed of two separate domains. Our structure reveals striking differences between the positions of INTS4 and Symplekin with respect to their nuclease heterodimer partners (Figures 7F–7H). Both proteins interact mainly via their NTDs with corresponding pseudonucleases (INTS9 or CPSF100) but are located on the opposite side of each complex. The CTD of INTS4 is poorly resolved in our map but identified unambiguously near the β-CASP domain of INTS9. An equivalent CTD domain of Symplekin binds the CPSF73/100 dimer at a very distant CPSF73/100 CTD2 dimerization domain (Zhang et al., 2020b). Such a differential binding mode is unexpected, given the high similarity of the nuclease heterodimer structures. This raises the question whether INTS4 and Symplekin are indeed functionally related. It is likely that the differences observed here are the result of a specialization of each machinery developed to recognize and process different sets of substrates. However, in principle, other INTSs could bind INTS9/11 in a manner resembling Symplekin binding, even in the presence of INTS4. Also, it cannot be excluded that each machinery could undergo a conformational rearrangement during assembly, and the two structures compared here may not represent the same functional state.

Our analysis revealed that INTS4/9/11 forms a highly electropositive composite groove leading toward the active site of INTS11, strongly suggesting a possible path for the RNA substrate within the complex (Figure 6). Currently, it is not clear whether this channel would accommodate a substrate upstream or downstream of the cleavage site because, in principle, both modes of binding could be supported. Importantly, such a binding mode would be very different from the one observed for the histone processing machinery, where $\text{Symplekin}^{\text{NTD}}$ forms an RNA-binding cavity on the opposite side of the nuclease heterodimer. The differences observed here may represent how the specialization necessary to accommodate different substrates or substrate recognition modules is achieved. However, despite the very different architecture of each complex, we recognize that the design principles of both machineries have some important similarities. Nuclease dimer formation and recruitment of two unrelated helical proteins may functionally play analogous roles in creating a substrate binding cavity. Functional characterization of additional modules identified in this work, in particular identification of the substrate binding module and the factors required to trigger nuclease activity, will be crucial to understand the underlying mechanism and the specificity of the Integrator complex.

While this manuscript was under review, a structure of the integrator-containing PP2A-AC complex (INTAC) was reported (Zheng et al., 2020), providing new insights into the architecture of a nearly complete Integrator complex and its function as a non-canonical RNAPII phosphatase. This new structure comprises two of the modules discussed in this manuscript, INTS5/8 and INTS4/9/11, which localize to opposite sides of the complex and do not interact, consistent with our biochemical data. INTS10/13/14 is present within the INTAC complex but not resolved in the cryo-EM map. INTS4/9/11, described by Zheng et al. (2020), shows overall good agreement with our structure (RMSD of 2.2 Å over 5,400 atoms), with only minor discrepancies in the amino acid register in the middle part of INTS4 (residues 280–340). The atomic coordinates of $\text{INTS4}^{\text{CTD}}$ can be readily fitted into the density attributed to this domain in our map. Interestingly, INTS11 remains in an inactive state despite numerous other components present in the complex, and its nuclease activation mechanism remains unknown.

Limitations of study

Our tandem affinity purification approach was designed to detect abundant sub-complexes containing unique components. Low-abundant INTSs, which were not enriched sufficiently in our analysis, might bridge different modules *in vivo*. Therefore, we cannot exclude the possibility that each of the modules described here could also exists within a larger assembly.

In addition, the nature of the control in our pull-down experiment (INTS4) implies that subunits shared between different sub-complexes would be well abundant but not enriched by either bait protein.

The RNA-binding properties of all three modules were tested *in vitro* on a model substrate. Although we observed reproducible and consistent formation of an RNP complex between INTS4/9/11 and the model substrate, we cannot prove that this binding is mediated by the electropositive patch identified as a putative RNA-binding surface. Mutagenesis of this surface and an *in vivo* assay (Figure 6) suggest that this surface is functionally important; however, INTS4 residues with the strongest misprocessing effect are also partially involved in binding of INTS9, and their mutation may have a more convoluted effect.

STAR★METHODS

Detailed methods are provided in the online version of this paper and include the following:

- KEY RESOURCES TABLE
- RESOURCE AVAILABILITY
  - Lead contact
  - Material availability

- Data and code availability
- EXPERIMENTAL MODEL AND SUBJECT DETAILS
- METHOD DETAILS
  - Protein expression and purification
  - Crosslinking gradient (GraFix)
  - Size exclusion chromatography
  - Sample vitrification
  - Cryo-EM data collection
  - Cryo-EM data processing
  - Model Building and Refinement
  - Cross-linking Mass spectrometry
  - Analytical pull-down and quantitative mass spectrometry
  - RNA *in vitro* transcription and labeling
  - EMSA
  - Dot blot
  - GFP-based *in vivo* reporter assay
  - Immunoprecipitation assay
  - Western blot
- QUANTIFICATION AND STATISTICAL ANALYSIS

SUPPLEMENTAL INFORMATION

Supplemental Information can be found online at https://doi.org/10.1016/j.molcel.2021.01.005.

ACKNOWLEDGMENTS

We acknowledge the EMBL Proteomic Core Facility, in particular Mandy Rettel, Per Haberkant, and Frank Stein, for assistance with mass spectrometry data acquisition and analysis; Felix Weis and Michael Hons for assistance with cryo-EM data collection at the EMBL cryo-EM platform and ESRF CM01 beamline; Estelle Marchal for technical assistance with RNA preparation; Erin Cutts and Alessandro Vannini for advice regarding the biGBac system; the EMBL Grenoble EEF platform for assistance with cell culture; and Lori Passmore, Daniel Peter, Stephen Cusack, Sagar Bhogaraju, and Michal Razew for critical comments on the manuscript.

AUTHOR CONTRIBUTIONS

M.M.P. identified, expressed, and purified stable Integrator sub-complexes, and performed cryo-EM data analysis, model building and refinement, EMSA assays, and pull-down validation. W.P.G. initiated and coordinated the project and provided support with experimental procedures. M.M.P. and W.P.G. analyzed the data and wrote the manuscript.

DECLARATION OF INTERESTS

The authors declare no competing interests.

Received: August 4, 2020
Revised: November 29, 2020
Accepted: January 5, 2021
Published: February 5, 2021

REFERENCES

Albrecht, T.R., and Wagner, E.J. (2012). snRNA 3′ end formation requires heterodimeric association of integrator subunits. Mol. Cell. Biol. *32*, 1112–1123.

Albrecht, T.R., Shevtsov, S.P., Wu, Y., Mascibroda, L.G., Peart, N.J., Huang, K.-L., Sawyer, I.A., Tong, L., Dundr, M., and Wagner, E.J. (2018). Integrator subunit 4 is a ‘Symplekin-like’ scaffold that associates with INTS9/11 to form the Integrator cleavage module. Nucleic Acids Res. *46*, 4241–4255.

Andrade, M.A., Petosa, C., O’Donoghue, S.I., Müller, C.W., and Bork, P. (2001). Comparison of ARM and HEAT protein repeats. J. Mol. Biol. *309*, 1–18.

Bai, X.-C., Rajendra, E., Yang, G., Shi, Y., and Scheres, S.H.W. (2015). Sampling the conformational space of the catalytic subunit of human γ-secretase. eLife *4*, 1485.

Baillat, D., and Wagner, E.J. (2015). Integrator: surprisingly diverse functions in gene expression. Trends Biochem. Sci. *40*, 257–264.

Baillat, D., Hakimi, M.-A., Näär, A.M., Shilatifard, A., Cooch, N., and Shiekhattar, R. (2005). Integrator, a multiprotein mediator of small nuclear RNA processing, associates with the C-terminal repeat of RNA polymerase II. Cell *123*, 265–276.

Barbieri, E., Trizzino, M., Welsh, S.A., Owens, T.A., Calabretta, B., Carroll, M., Sarma, K., and Gardini, A. (2018). Targeted Enhancer Activation by a Subunit of the Integrator Complex. Mol. Cell *71*, 103–116.e7.

Becher, I., Andrés-Pons, A., Romanov, N., Stein, F., Schramm, M., Baudin, F., Helm, D., Kurzawa, N., Mateus, A., Mackmull, M.-T., et al. (2018). Pervasive Protein Thermal Stability Variation during the Cell Cycle. Cell *173*, 1495–1507.e18.

Beckedorff, F., Blumenthal, E., daSilva, L.F., Aoi, Y., Cingaram, P.R., Yue, J., Zhang, A., Dokaneheifard, S., Valencia, M.G., Gaidosh, G., et al. (2020). The Human Integrator Complex Facilitates Transcriptional Elongation by Endonucleolytic Cleavage of Nascent Transcripts. Cell Rep. *32*, 107917.

Bieniossek, C., Imasaki, T., Takagi, Y., and Berger, I. (2012). MultiBac: expanding the research toolbox for multiprotein complexes. Trends Biochem. Sci. *37*, 49–57.

Bienroth, S., Keller, W., and Wahle, E. (1993). Assembly of a processive messenger RNA polyadenylation complex. EMBO J. *12*, 585–594.

Brown, A., Long, F., Nicholls, R.A., Toots, J., Emsley, P., and Murshudov, G. (2015). Tools for macromolecular model building and refinement into electron cryo-microscopy reconstructions. Acta Crystallogr. D Biol. Crystallogr. *71*, 136–153.

Burnley, T., Palmer, C.M., and Winn, M. (2017). Recent developments in the CCP-EM software suite. Acta Crystallogr. D Struct. Biol. *73*, 469–477.

Casañal, A., Kumar, A., Hill, C.H., Easter, A.D., Emsley, P., Degliesposti, G., Gordiyenko, Y., Santhanam, B., Wolf, J., Wiederhold, K., et al. (2017). Architecture of eukaryotic mRNA 3′-end processing machinery. Science *358*, 1056–1059.

Cazalla, D., Xie, M., and Steitz, J.A. (2011). A primate herpesvirus uses the integrator complex to generate viral microRNAs. Mol. Cell *43*, 982–992.

Chen, J., Ezzeddine, N., Waltenspiel, B., Albrecht, T.R., Warren, W.D., Marzluff, W.F., and Wagner, E.J. (2012). An RNAi screen identifies additional members of the Drosophila Integrator complex and a requirement for cyclin C/Cdk8 in snRNA 3′-end formation. RNA *18*, 2148–2156.

Ciliberto, G., Dathan, N., Frank, R., Philipson, L., and Mattaj, I.W. (1986). Formation of the 3′ end on U snRNAs requires at least three sequence elements. EMBO J. *5*, 2931–2937.

Clerici, M., Faini, M., Muckenfuss, L.M., Aebersold, R., and Jinek, M. (2018). Structural basis of AAUAAA polyadenylation signal recognition by the human CPSF complex. Nat. Struct. Mol. Biol. *25*, 135–138.

Davidson, L., Francis, L., Eaton, J.D., and West, S. (2020). Integrator-Dependent and Allosteric/Intrinsic Mechanisms Ensure Efficient Termination of snRNA Transcription. Cell Rep. *33*, 108319.

Dignam, J.D., Lebovitz, R.M., and Roeder, R.G. (1983). Accurate transcription initiation by RNA polymerase II in a soluble extract from isolated mammalian nuclei. Nucleic Acids Res. *11*, 1475–1489.

Dominski, Z., Yang, X.-C., and Marzluff, W.F. (2005a). The polyadenylation factor CPSF-73 is involved in histone-pre-mRNA processing. Cell *123*, 37–48.

Dominski, Z., Yang, X.C., Purdy, M., Wagner, E.J., and Marzluff, W.F. (2005b). A CPSF-73 homologue is required for cell cycle progression but not cell growth and interacts with a protein having features of CPSF-100. Mol. Cell. Biol. *25*, 1489–1500.

Dominski, Z., Carpousis, A.J., and Clouet-d'Orval, B. (2013). Emergence of the β-CASP ribonucleases: Highly conserved and ubiquitous metallo-enzymes involved in messenger RNA maturation and degradation. Biochim. Biophys. Acta *1829*, 532–551.

Dorléans, A., Li de la Sierra-Gallay, I., Piton, J., Zig, L., Gilet, L., Putzer, H., and Condon, C. (2011). Molecular basis for the recognition and cleavage of RNA by the bifunctional 5′-3′ exo/endoribonuclease RNase J. Structure *19*, 1252–1261.

Egloff, S., O'Reilly, D., Chapman, R.D., Taylor, A., Tanzhaus, K., Pitts, L., Eick, D., and Murphy, S. (2007). Serine-7 of the RNA polymerase II CTD is specifically required for snRNA gene expression. Science *318*, 1777–1779.

Elrod, N.D., Henriques, T., Huang, K.-L., Tatomer, D.C., Wilusz, J.E., Wagner, E.J., and Adelman, K. (2019). The Integrator Complex Attenuates Promoter-Proximal Transcription at Protein-Coding Genes. Mol. Cell *76*, 738–752.e7.

Emsley, P., and Cowtan, K. (2004). Coot: model-building tools for molecular graphics. Acta Crystallogr. D Biol. Crystallogr. *60*, 2126–2132.

Gardini, A., Baillat, D., Cesaroni, M., Hu, D., Marinis, J.M., Wagner, E.J., Lazar, M.A., Shilatifard, A., and Shiekhattar, R. (2014). Integrator regulates transcriptional initiation and pause release following activation. Mol. Cell *56*, 128–139.

Goddard, T.D., Huang, C.C., Meng, E.C., Pettersen, E.F., Couch, G.S., Morris, J.H., and Ferrin, T.E. (2018). UCSF ChimeraX: Meeting modern challenges in visualization and analysis. Protein Sci. *27*, 14–25.

Hardin, J.W., Warnasooriya, C., Kondo, Y., Nagai, K., and Rueda, D. (2015). Assembly and dynamics of the U4/U6 di-snRNP by single-molecule FRET. Nucleic Acids Res. *43*, 10963–10974.

Hernandez, N. (1985). Formation of the 3′ end of U1 snRNA is directed by a conserved sequence located downstream of the coding region. EMBO J. *4*, 1827–1837.

Hernandez, N., and Weiner, A.M. (1986). Formation of the 3′ end of U1 snRNA requires compatible snRNA promoter elements. Cell *47*, 249–258.

Hill, C.H., Boreikaitė, V., Kumar, A., Casañal, A., Kubík, P., Degliesposti, G., Maslen, S., Mariani, A., von Loeffelholz, O., Girbig, M., et al. (2019). Activation of the Endonuclease that Defines mRNA 3′ Ends Requires Incorporation into an 8-Subunit Core Cleavage and Polyadenylation Factor Complex. Mol. Cell *73*, 1217–1231.e11.

Huang, K.-L., Jee, D., Stein, C.B., Elrod, N.D., Henriques, T., Mascibroda, L.G., Baillat, D., Russell, W.K., Adelman, K., and Wagner, E.J. (2020). Integrator Recruits Protein Phosphatase 2A to Prevent Pause Release and Facilitate Transcription Termination. Mol. Cell *80*, 345–358.e9.

Huber, W., von Heydebreck, A., Sültmann, H., Poustka, A., and Vingron, M. (2002). Variance stabilization applied to microarray data calibration and to the quantification of differential expression. Bioinformatics *18* (*Suppl 1*), S96–S104.

Hughes, C.S., Foehr, S., Garfield, D.A., Furlong, E.E., Steinmetz, L.M., and Krijgsveld, J. (2014). Ultrasensitive proteome analysis using paramagnetic bead technology. Mol. Syst. Biol. *10*, 757.

Jia, Y., Cheng, Z., Bharath, S.R., Sun, Q., Su, N., Huang, J., and Song, H. (2020). Crystal Structure of INTS3/INTS6 complex reveals Functional Importance of INTS3 dimerization in DSB Repair. bioRxiv. https://doi.org/10.1101/2020.04.10.035014.

Kandiah, E., Giraud, T., de Maria Antolinos, A., Dobias, F., Effantin, G., Flot, D., Hons, M., Schoehn, G., Susini, J., Svensson, O., et al. (2019). CM01: a facility for cryo-electron microscopy at the European Synchrotron. Acta Crystallogr. D Struct. Biol. *75*, 528–535.

Kastner, B., Fischer, N., Golas, M.M., Sander, B., Dube, P., Boehringer, D., Hartmuth, K., Deckert, J., Hauer, F., Wolf, E., et al. (2008). GraFix: sample preparation for single-particle electron cryomicroscopy. Nat. Methods *5*, 53–55.

Kelley, L.A., Mezulis, S., Yates, C.M., Wass, M.N., and Sternberg, M.J.E. (2015). The Phyre2 web portal for protein modeling, prediction and analysis. Nat. Protoc. *10*, 845–858.

Kolev, N.G., and Steitz, J.A. (2005). Symplekin and multiple other polyadenylation factors participate in 3′-end maturation of histone mRNAs. Genes Dev. *19*, 2583–2592.

Krall, M., Htun, S., Schnur, R.E., Brooks, A.S., Baker, L., de Alba Campomanes, A., Lamont, R.E., Gripp, K.W., Schneidman-Duhovny, D., Innes, A.M., et al.; Care 4 Rare Canada Consortium (2019). Biallelic sequence variants in INTS1 in patients with developmental delays, cataracts, and craniofacial anomalies. Eur. J. Hum. Genet. *27*, 582–593.

Lai, F., Gardini, A., Zhang, A., and Shiekhattar, R. (2015). Integrator mediates the biogenesis of enhancer RNAs. Nature *525*, 399–403.

Lobo, S.M., and Hernandez, N. (1989). A 7 bp mutation converts a human RNA polymerase II snRNA promoter into an RNA polymerase III promoter. Cell *58*, 55–67.

Lykke-Andersen, S., Žumer, K., Molska, E.Š., Rouvière, J.O., Wu, G., Demel, C., Schwalb, B., Schmid, M., Cramer, P., and Jensen, T.H. (2020). Integrator is a genome-wide attenuator of non-productive transcription. Mol. Cell. Published online December 23, 2020. https://doi.org/10.1016/j.molcel.2020.12.014.

Mandel, C.R., Kaneko, S., Zhang, H., Gebauer, D., Vethantham, V., Manley, J.L., and Tong, L. (2006). Polyadenylation factor CPSF-73 is the pre-mRNA 3′-end-processing endonuclease. Nature *444*, 953–956.

Mascibroda, L.G., Shboul, M., Elrod, N.D., Colleaux, L., Hamamy, H., Huang, K.-L., Peart, N., Singh, M.K., Lee, H., Merriman, B., et al. (2020). INTS13 Mutations Causing a Developmental Ciliopathy Disrupt Integrator Complex Assembly. bioRxiv. https://doi.org/10.1101/2020.07.20.209130.

Mastronarde, D.N. (2005). Automated electron microscope tomography using robust prediction of specimen movements. J. Struct. Biol. *152*, 36–51.

Michalski, D., and Steiniger, M. (2015). In vivo characterization of the Drosophila mRNA 3′ end processing core cleavage complex. RNA *21*, 1404–1418.

Milligan, J.F., Groebe, D.R., Witherell, G.W., and Uhlenbeck, O.C. (1987). Oligoribonucleotide synthesis using T7 RNA polymerase and synthetic DNA templates. Nucleic Acids Res. *15*, 8783–8798.

Moggridge, S., Sorensen, P.H., Morin, G.B., and Hughes, C.S. (2018). Extending the Compatibility of the SP3 Paramagnetic Bead Processing Approach for Proteomics. J. Proteome Res. *17*, 1730–1740.

Mowry, K.L., and Steitz, J.A. (1987). Identification of the human U7 snRNP as one of several factors involved in the 3′ end maturation of histone premessenger RNA's. Science *238*, 1682–1687.

Murshudov, G.N., Skubák, P., Lebedev, A.A., Pannu, N.S., Steiner, R.A., Nicholls, R.A., Winn, M.D., Long, F., and Vagin, A.A. (2011). REFMAC5 for the refinement of macromolecular crystal structures. Acta Crystallogr. D Biol. Crystallogr. *67*, 355–367.

Murthy, K.G., and Manley, J.L. (1992). Characterization of the multisubunit cleavage-polyadenylation specificity factor from calf thymus. J. Biol. Chem. *267*, 14804–14811.

Nicholls, R.A., Fischer, M., McNicholas, S., and Murshudov, G.N. (2014). Conformation-independent structural comparison of macromolecules with ProSMART. Acta Crystallogr. D Biol. Crystallogr. *70*, 2487–2499.

Nojima, T., Tellier, M., Foxwell, J., Ribeiro de Almeida, C., Tan-Wong, S.M., Dhir, S., Dujardin, G., Dhir, A., Murphy, S., and Proudfoot, N.J. (2018). Deregulated Expression of Mammalian lncRNA through Loss of SPT6 Induces R-Loop Formation, Replication Stress, and Cellular Senescence. Mol. Cell *72*, 970–984.e7.

Oegema, R., Baillat, D., Schot, R., van Unen, L.M., Brooks, A., Kia, S.K., Hoogeboom, A.J.M., Xia, Z., Li, W., Cesaroni, M., et al. (2017). Human mutations in integrator complex subunits link transcriptome integrity to brain development. PLoS Genet. *13*, e1006809.

Pei, J., Tang, M., and Grishin, N.V. (2008). PROMALS3D web server for accurate multiple protein sequence and structure alignments. Nucleic Acids Res. *36*, W30-4.

CellPress
OPEN ACCESS

Pettinati, I., Brem, J., Lee, S.Y., McHugh, P.J., and Schofield, C.J. (2016). The Chemical Biology of Human Metallo-β-Lactamase Fold Proteins. Trends Biochem. Sci. *41*, 338–355.

Pillai, R.S., Grimmler, M., Meister, G., Will, C.L., Lührmann, R., Fischer, U., and Schümperli, D. (2003). Unique Sm core structure of U7 snRNPs: assembly by a specialized SMN complex and the role of a new component, Lsm11, in histone RNA processing. Genes Dev. *17*, 2321–2333.

Preker, P.J., Ohnacker, M., Minvielle-Sebastia, L., and Keller, W. (1997). A multisubunit 3′ end processing factor from yeast containing poly(A) polymerase and homologues of the subunits of mammalian cleavage and polyadenylation specificity factor. EMBO J. *16*, 4727–4737.

Proudfoot, N.J. (2011). Ending the message: poly(A) signals then and now. Genes Dev. *25*, 1770–1782.

Rienzo, M., and Casamassimi, A. (2016). Integrator complex and transcription regulation: Recent findings and pathophysiology. Biochim. Biophys. Acta *1859*, 1269–1280.

Rohou, A., and Grigorieff, N. (2015). CTFFIND4: Fast and accurate defocus estimation from electron micrographs. J. Struct. Biol. *192*, 216–221.

Rubtsova, M.P., Vasilkova, D.P., Moshareva, M.A., Malyavko, A.N., Meerson, M.B., Zatsepin, T.S., Naraykina, Y.V., Beletsky, A.V., Ravin, N.V., and Dontsova, O.A. (2019). Integrator is a key component of human telomerase RNA biogenesis. Sci. Rep. *9*, 1701–1710.

Ryder, S.P., Recht, M.I., and Williamson, J.R. (2008). Quantitative analysis of protein-RNA interactions by gel mobility shift. Methods Mol. Biol. *488*, 99–115.

Sabath, K., Stäubli, M.L., Marti, S., Leitner, A., Moes, M., and Jonas, S. (2020). INTS10-INTS13-INTS14 form a functional module of Integrator that binds nucleic acids and the cleavage module. Nat. Commun. *11*, 3422.

Savitski, M.M., Wilhelm, M., Hahne, H., Kuster, B., and Bantscheff, M. (2015). A Scalable Approach for Protein False Discovery Rate Estimation in Large Proteomic Data Sets. Mol. Cell. Proteomics *14*, 2394–2404.

Scheres, S.H.W. (2012). RELION: implementation of a Bayesian approach to cryo-EM structure determination. J. Struct. Biol. *180*, 519–530.

Scheres, S.H.W., and Chen, S. (2012). Prevention of overfitting in cryo-EM structure determination. Nat. Methods *9*, 853–854.

Shi, Y., and Manley, J.L. (2015). The end of the message: multiple protein-RNA interactions define the mRNA polyadenylation site. Genes Dev. *29*, 889–897.

Skaar, J.R., Richard, D.J., Saraf, A., Toschi, A., Bolderson, E., Florens, L., Washburn, M.P., Khanna, K.K., and Pagano, M. (2009). INTS3 controls the hSSB1-mediated DNA damage response. J. Cell Biol. *187*, 25–32.

Skaar, J.R., Ferris, A.L., Wu, X., Saraf, A., Khanna, K.K., Florens, L., Washburn, M.P., Hughes, S.H., and Pagano, M. (2015). The Integrator complex controls the termination of transcription at diverse classes of gene targets. Cell Res. *25*, 288–305.

Sullivan, K.D., Steiniger, M., and Marzluff, W.F. (2009). A core complex of CPSF73, CPSF100, and Symplekin may form two different cleavage factors for processing of poly(A) and histone mRNAs. Mol. Cell *34*, 322–332.

Sun, Y., Zhang, Y., Hamilton, K., Manley, J.L., Shi, Y., Walz, T., and Tong, L. (2018). Molecular basis for the recognition of the human AAUAAA polyadenylation signal. Proc. Natl. Acad. Sci. USA *115*, E1419–E1428.

Sun, Y., Zhang, Y., Aik, W.S., Yang, X.-C., Marzluff, W.F., Walz, T., Dominski, Z., and Tong, L. (2020). Structure of an active human histone pre-mRNA 3′-end processing machinery. Science *367*, 700–703.

Swale, C., Bougdour, A., Gnahoui-David, A., Tottey, J., Georgeault, S., Laurent, F., Palencia, A., and Hakimi, M.-A. (2019). Metal-captured inhibition of pre-mRNA processing activity by CPSF3 controls *Cryptosporidium* infection. Sci. Transl. Med. *11*, eaax7161.

Tatomer, D.C., Elrod, N.D., Liang, D., Xiao, M.-S., Jiang, J.Z., Jonathan, M., Huang, K.-L., Wagner, E.J., Cherry, S., and Wilusz, J.E. (2019). The Integrator complex cleaves nascent mRNAs to attenuate transcription. Genes Dev. *33*, 1525–1538.

Weissmann, F., Petzold, G., VanderLinden, R., Huis In 't Veld, P.J., Brown, N.G., Lampert, F., Westermann, S., Stark, H., Schulman, B.A., and Peters, J.M. (2016). biGBac enables rapid gene assembly for the expression of large multisubunit protein complexes. Proc. Natl. Acad. Sci. USA *113*, E2564–E2569.

Werner, T., Sweetman, G., Savitski, M.F., Mathieson, T., Bantscheff, M., and Savitski, M.M. (2014). Ion coalescence of neutron encoded TMT 10-plex reporter ions. Anal. Chem. *86*, 3594–3601.

Wilkinson, M.E., Kumar, A., and Casañal, A. (2019). Methods for merging data sets in electron cryo-microscopy. Acta Crystallogr. D Struct. Biol. *75*, 782–791.

Wu, Y., Albrecht, T.R., Baillat, D., Wagner, E.J., and Tong, L. (2017). Molecular basis for the interaction between Integrator subunits IntS9 and IntS11 and its functional importance. Proc. Natl. Acad. Sci. USA *114*, 4394–4399.

Xie, M., Zhang, W., Shu, M.-D., Xu, A., Lenis, D.A., DiMaio, D., and Steitz, J.A. (2015). The host Integrator complex acts in transcription-independent maturation of herpesvirus microRNA 3′ ends. Genes Dev. *29*, 1552–1564.

Yamamoto, J., Hagiwara, Y., Chiba, K., Isobe, T., Narita, T., Handa, H., and Yamaguchi, Y. (2014). DSIF and NELF interact with Integrator to specify the correct post-transcriptional fate of snRNA genes. Nat. Commun. *5*, 4263.

Yuo, C.Y., Ares, M., Jr., and Weiner, A.M. (1985). Sequences required for 3′ end formation of human U2 small nuclear RNA. Cell *42*, 193–202.

Zhang, Y. (2008). I-TASSER server for protein 3D structure prediction. BMC Bioinformatics *9*, 40–48.

Zhang, F., Ma, T., and Yu, X. (2013). A core hSSB1-INTS complex participates in the DNA damage response. J. Cell Sci. *126*, 4850–4855.

Zhang, X., Wang, Y., Yang, F., Tang, J., Xu, X., Yang, L., Yang, X.-A., and Wu, D. (2020a). Biallelic INTS1 Mutations Cause a Rare Neurodevelopmental Disorder in Two Chinese Siblings. J. Mol. Neurosci. *70*, 1–8.

Zhang, Y., Sun, Y., Shi, Y., Walz, T., and Tong, L. (2020b). Structural Insights into the Human Pre-mRNA 3′-End Processing Machinery. Mol. Cell *77*, 800–809.e6.

Zheng, S.Q., Palovcak, E., Armache, J.-P., Verba, K.A., Cheng, Y., and Agard, D.A. (2017). MotionCor2: anisotropic correction of beam-induced motion for improved cryo-electron microscopy. Nat. Methods *14*, 331–332.

Zheng, H., Qi, Y., Hu, S., Cao, X., Xu, C., Yin, Z., Chen, X., Li, Y., Liu, W., Li, J., et al. (2020). Identification of Integrator-PP2A complex (INTAC), an RNA polymerase II phosphatase. Science *370*, eabb5872.

Zivanov, J., Nakane, T., Forsberg, B.O., Kimanius, D., Hagen, W.J., Lindahl, E., and Scheres, S.H. (2018). New tools for automated high-resolution cryo-EM structure determination in RELION-3. eLife *7*, 163.

Zivanov, J., Nakane, T., and Scheres, S.H.W. (2019). A Bayesian approach to beam-induced motion correction in cryo-EM single-particle analysis. IUCrJ *6*, 5–17.

STAR★METHODS

KEY RESOURCES TABLE

REAGENT or RESOURCE	SOURCE	IDENTIFIER
Antibodies		
Mouse anti-FLAG (HRP conjugate)	Sigma	Cat#A8592; RRID:AB_439702
Rabbit anti-INTS4	Abcam	Cat#ab75253; RRID:AB_1280962
Rabbit anti-INTS11	Cusabio	Cat#PA722574ESR1HU; RRID:AB_2888982
Rabbit anti-GAPDH	ProteinTech	Cat#10494-1-AP; RRID:AB_2263076
Mouse anti-SBP	EMD Millipore	Cat#MAB10764; RRID:AB_10631872
Goat anti-Rabbit	Abcam	Cat#ab205718; RRID:AB_2819160
Mouse anti-HA (HRP-Conjugate)	Santa Cruz	Cat#SC7392HRP; RRID:AB_627809
Goat anti-Mouse	Thermo Fisher	Cat#31430; RRID:AB_228307
Bacterial and virus strains		
NEB10	NEB	Cat# C3020K
DH10EMBacY	Bieniossek et al., 2012	N/A
Chemicals, peptides, and recombinant proteins		
RNAiMAX	Invitrogen	Cat#13778030
L-Glutamine	GIBCO	Cat#A29168-01
Penicillin-Streptomycin (10,000 U/mL)	GIBCO	Cat#15140-122
DMEM	GIBCO	Cat#31966-021
ExpressFive™ SFM	GIBCO	Cat#10486025
SF-900™ II SFM	GIBCO	Cat#11497013
Opti-MEM	GIBCO	Cat#31985-062
Trypsin-EDTA	GIBCO	Cat#25200-056
FBS	GIBCO	Cat#10270-106
LipoD293	Sinagen	Cat#SL100668
PEI 25k	Polysciences	Cat#23966-1
Tween20	Sigma	Cat#P1379-500ML
Freestyle™ 293 Expression Medium	GIBCO	Cat#12338018
Glutaraldehyde	Sigma Aldrich	Cat#G5882-10X1ML
Triton X-100	ICN Biomedicals	Cat#807426
Pierce™ ECL Western Blotting Substrate	Thermo Fisher	Cat#32209
Immobilon Membrane	Merck	Cat#PVH000010
Anti-FLAG Resin	Sigma Aldrich	Cat#F2426-1ML
Anti-HA Resin	Sigma Aldrich	Cat#A2905-1ML
High Capacity Streptavidin Agarose Resin	Thermo Fisher	Cat#20361
His60 Ni Superflow Resin	Takara	Cat#635660
IgG Sepharose™ 6 Fast Flow	GE Healthcare	Cat#17-0969-01
Amylose Resin	NEB	Cat#E8021S
Deposited data		
High-resolution cryo-EM map	This paper	EMDB: EMD-12165
Medium-resolution cryo-EM map	This paper	EMDB: EMD-12166
INTS4-focused cryo-EM map	This paper	EMDB: EMD-12164
CTD2-focused cryo-EM map	This paper	EMDB: EMD-12163
Model refined against high-resolution map	This paper	PDB: 7BFP
Pseudoatomic model fitted into medium-resolution map	This paper	PDB: 7BFQ

(Continued on next page)

CellPress
OPEN ACCESS

Continued

REAGENT or RESOURCE	SOURCE	IDENTIFIER
**Experimental models: cell lines**		
HEK293T	ATCC	Cat#CRL-3216
FreeStyle^{Tm} 293-F	GIBCO	Cat#R79007
SF21	GIBCO	Cat#11497013
Trichoplusia ni High Five Cells	Invitrogen	Cat#B85502
**Oligonucleotides**		
INTS4 siRNA (5′-GUAGGCUUAAGGAGUAUGUGAUU-3′)	Dharmacon, based on Albrecht et al. (2018)	N/A
INTS11 siRNA (5′-CAGACUUCCUGGACUGUGUUU-3′)	Dharmacon, based on Albrecht et al. (2018)	N/A
Scrambled siRNA control (5′-UGCACCGAGUGGCGACACCUU-3′)	this study	N/A
**Recombinant DNA**		
pBIG1a+8xHis-INTS4/INTS9/SBP-INTS11	this study	N/A
pBIG1a+8xHis-INTS5/SBP-INTS8	this study	N/A
pBIG1c+8xHis-INTS10/INTS13/SBP-INTS14	this study	N/A
pMG-3xFLAG+INTS11	this study	N/A
pMG-3xFLAG+INTS11$^{\Delta CTD2}$	this study	N/A
pMG-3xFLAG+INTS11$^{\Delta CTD1 \Delta CTD2}$	this study	N/A
pMG-3xFLAG+INTS11$^{\Delta CTD1}$	this study	N/A
pMG_3xFLAG+INTS11$^{H480P/T482P}$	this study	N/A
pMG-3xFLAG+INTS11$^{V454P/G455P}$	this study	N/A
pMG-3xFLAG+INTS11$^{F490P/L492P}$	this study	N/A
pMG-3xFLAG+INTS11^{K462E}	this study	N/A
pMG-3xFLAG+INTS11$^{R510P/T512P}$	based on Wu et al. (2017)	N/A
pMG-3xFLAG+INTS11^{E203Q}	based on Baillat et al. (2005)	N/A
pMG-SBP+INTS4$^{K122A/H124A/Q125A}$	this study	N/A
pMG-SBP+INTS4$^{H164A/R167A}$	this study	N/A
pMG-SBP+INTS4^{R210A}	this study	N/A
**Software and algorithms**		
PyMol	N/A	https://pymol.org/2/
Coot	Emsley and Cowtan, 2004	https://www2.mrc-lmb.cam.ac.uk/personal/pemsley/coot/
CCPEM	Burnley et al., 2017	https://www.ccpem.ac.uk/
Relion	Scheres, 2012	https://www3.mrc-lmb.cam.ac.uk/relion/
UCSF ChimeraX	Goddard et al., 2018	https://www.cgl.ucsf.edu/chimerax/
Image Lab	Bio-Rad	http://www.bio-rad.com/en-fr/product/image-lab-software?ID=KRE6P5E8Z
Fiji		https://imagej.net/Fiji
MARS	BMG Labtech	https://www.bmglabtech.com/de/mars-datenanalyse-software/
Promals3D	Pei et al., 2008	http://prodata.swmed.edu/promals3d/promals3d.php
**Other**		
UltrAuFoil R1.2/1.3 300 mesh	Quantifoil	Cat#Q350AR13A
Superose 6 3.2/300 Increase	GE Healthcare	Cat#GE29-0915-98
Vitribot Mark IV	Thermo Fisher Scientific	https://www.thermofisher.com/us/en/home.html

(*Continued on next page*)

Continued		
REAGENT or RESOURCE	SOURCE	IDENTIFIER
Amicon-Ultra 0.5 ml Centrifugal filters – 50 kDa	Merck	UFC5050
96-well plate, black	Corning	Cat#3925
CLARIOstar	BMG Labtech	https://www.bmglabtech.com/clariostar-plus/
ChemiDoc MP	Bio-Rad	http://www.bio-rad.com/en-us/category/chemidoc-imaging-systems?ID=NINJ0Z15
Vibra Cell VCX750	Sonics	https://www.sonics.com/liquid-processing/products/vibra-cell-processors/vcx-500-vcx-750/

RESOURCE AVAILABILITY

Lead contact

Further information and requests for resources and reagents should be directed to and will be fulfilled by the Lead Contact, Wojciech P. Galej (wgalej@embl.fr).

Material availability

Unique and stable reagents generated in this study are available upon request.

Data and code availability

Cryo-EM maps obtained within this project have been deposited in the EMDB database with the following accession codes: EMD-12165 (High-resolution map), EMD-12166 (Medium-resolution, ESRF1 map), EMD-12164 (INTS4-focused map), EMD-12163 (CTD2-focused map). The atomic coordinates have been deposited in PDBe with the following accession codes: 7BFP (model refined against the high-resolution map) and 7BFQ (pseudoatomic model fitted into the medium-resolution map).

EXPERIMENTAL MODEL AND SUBJECT DETAILS

HEK293T cells (ATCC) were propagated in DMEM medium (GIBCO) supplemented with 10% FBS (Thermo Fisher) and Pen Strep (GIBCO).

SF21 Insect cells were cultured in SF-900^{TM} II SFM media (GIBCO). Hi5 cells were grown in Express Five^{TM} SFM media (GIBCO) supplemented with L-Glutamine (GIBCO).

METHOD DETAILS

Protein expression and purification

Full-length open reading frames (ORFs) of INTS4 (N-terminally 8xHis-tagged), INTS9 and INTS11 (N-terminally SBP-tagged) were assembled into one vector using the biGBac system (Weissmann et al., 2016). Similarly, INTS5 (N-terminally 8xHis-tagged) and INTS8 (N-terminally SBP-tagged) as well as INTS10 (N-terminally 8xHis-tagged), INTS13 (N-terminally SBP-tagged) and INTS14 were cloned into a single vector containing two or three ORFs respectively. Baculovirus was generated in SF21 cells grown in SF-900^{TM} II SFM media, as previously described (Bieniossek et al., 2012). Hi5 cells, used for protein production, were grown in Express Five^{TM} SFM media and infected at a density of 1x10^{6} with 1% volume of the SF21 pre-culture and incubated for 72h at 27°C.

Cells were split into 500 mL aliquots, harvested at 300 g for 15 min at 4°C (JLA8.1000), resuspended in PBS and transferred to 50 mL tubes. The cells were spun down again at 500 g for 10 min (A-4-62), frozen in liquid nitrogen and stored at −80°C until further use.

The cell pellets were thawed in 10 volumes Buffer1 (150 mM KCl; 20 mM HEPES KOH pH 7,8; 30 mM Imidazole) and sonicated (4 times 1 min, 30% Amplitude, 10 s ON/OFF cycle). The membrane fraction was pelleted at 48.384 g for 45 min at 4°C (JA-25.50) and the supernatant incubated on 10% (v/v) Ni-NTA resin (QIAGEN) at 4°C for 2 h on a turning wheel. The resin was transferred to Poly-Prep Chromatography Columns (Bio-Rad) washed 5 times in 2 volumes Buffer1 and subsequently eluted in 5 × 1ml Buffer2 (150 mM KCl; 20 mM HEPES KOH pH 7.8; 250 mM Imidazole). The elutions were pooled, transferred to 200 μl Streptavidin agarose resin and again incubated for 2h at 4°C on a turning wheel. Subsequently, the resin was washed 5 times with 1 mL Buffer3 (150 mM KCl; 20 mM HEPES KOH pH 7.8) and eluted in 3 steps with Buffer4 (150 mM KCl; 20 mM HEPES KOH pH 7.8; 10 mM desthiobiotin).

Sample quality was monitored at the different stages by SDS-PAGE.

Crosslinking gradient (GraFix)

The elution fractions of the freshly purified complexes were pooled and transferred to a crosslinking gradient, GraFix (Kastner et al., 2008), with the following buffer composition: 10% - 30% Glycerol, 150 mM KCl, 20 mM HEPES KOH pH 7.8; 0,05% Glutaraldehyde and spun for 14 h at 160,000 g (SW60Ti). The gradient was aliquoted in 150 μl fractions, quenched with 20 mM Tris-HCl pH 7.8 (final concentration) and the protein complex traced with a Dot-Blot against the SBP-tag. Fractions containing cross-linked complex were pooled and the glycerol was removed by multiple rounds of concentration using an 0.5 mL Amicon spin column (50 kDa cut-off).

Size exclusion chromatography

50 μL of the freshly purified complexes at 1-2 mg/ml were injected into a Superose6® Increase 3.2/300 column and eluted over 3.3 mL equilibrated in Buffer3 at the flowrate of 0.04 ml/min. Fractions of 100 μL were collected and subsequently analyzed by SDS-PAGE.

Interactions were assessed by mixing the purified protein complexes in 1:1 molar ratio and incubating them on ice for 30 min before injections.

Sample vitrification

R1.2/1.3 UltrAuFoil 300 mesh grids were glow-discharged from each side for 20 s at 25 mA at 0.3 bar using a Pelco EasyGlow device. The protein concentration was adjusted to 0.5 mg/ml as described above and 2.5 μl sample applied to each side of the grid. Excess of the sample was blotted away using a Vitrobot MARK IV at 4°C, 100% humidity for 2 s at −10 blotting force and plunge frozen in liquid ethane.

Cryo-EM data collection

The grids were loaded into a Titan Krios (FEI) electron microscope at the CM01 ESRF beamline (Kandiah et al., 2019) (dataset ESRF1 and ESRF3) or at the EMBL Heidelberg cryo-EM platform (dataset EMBL2), both equipped with a K2 Summit direct electron detector and a GIF Quantum energy filter (Gatan). Microscopes were operated at 300kV acceleration voltage in an EF-TEM mode. The cryo-EM data was acquired using Thermo Fisher EPU software (ESRF) or serialEM (Mastronarde, 2005) (EMBL) at a nominal magnification of × 165 000, resulting in 0.83 $Å \cdot pixel^{-1}$ (ESRF) and 0.81 $Å \cdot pixel^{-1}$ (EMBL). ESRF movies were acquired for 4 s at a flux of 11.7 $e \cdot Å^{-2} \cdot s^{-1}$ and the total fluence of 46.8 $e \cdot Å^{-2}$ was fractionated into 40 movie frames. EMBL movies were acquired for 6 s at a flux of 7 $e \cdot Å^{-2} \cdot s^{-1}$ and the total fluence of 42 $e \cdot Å^{-2}$ was fractionated into 40 movie frames. A total of 6182 (ESRF) and 13,086 (EMBL) movies were acquired with a defocus range from −0.5 to −3.0 μm.

Cryo-EM data processing

All image processing was performed within Relion 3.0 (Zivanov et al., 2018), unless stated otherwise. For all three datasets beam-induced motion correction was performed using Relion's implementation of MotionCorr2 (Zheng et al., 2017) with a 5x5 patch model without binning followed by CTF estimation with CTFFIND 4.1 (Rohou and Grigorieff, 2015).

The datasets ESRF1 (1.02 million particles) and EMBL2 (4.65 million particles) were processed separately using a standard Relion workflow (Scheres, 2012), yielding 4.08 Å (118 k particles) and 3.98 Å (84 k particles) resolution respectively.

Automated particle picking of the ESRF1 dataset identified 1.02 million particles which were subjected to 3 rounds of 2D classification which reduced the particle number to 691 k. Two subsequent rounds of 3D classification with 3 classes each and a subsequent refinement resulted in a 8.3 Å resolution map with 302k particles. The refined map was classified further into 3 classes, the best class refined and again split into 5 classes. The best class containing 22 k particles was refined and yielded a 4.08 Å resolution map.

In case of the EMBL2 dataset automated picking in Relion identified 4.65 million particles. Due to the high number of particles, extraction was performed with 2-fold binning. The particles were subjected to 6 rounds of 2D classification to remove broken and poorly aligning particles, which reduced the particle number to 241 k. Those particles were re-extracted with their full pixel size and subjected to 3D classification into 6 classes. The best class, containing 84k particles, was refined and yielded a 3.98 Å resolution map.

The ESRF3 dataset was not processed separately as it was collected with the intention of merging the data with the previously collected datasets.

The final density of the EMBL2 dataset was used for template picking on all 3 datasets (ESRF1, EMBL2, ESRF2), yielding 2.9 million particles for the ESRF datasets and 6.1 million particles for the EMBL dataset. The ESRF particles were extracted from datasets 1 and 3 with full pixel size and merged. 2 rounds of 2D classification reduced the particle number to 786 k. The EMBL dataset was extracted 4-fold binned and subjected to 4 rounds of 2D classification which reduced the particle number to 1.6 million.

Since none of these datasets achieved the resolution required for *de novo* model building, we decided to merge all available data to improve the overall resolution. In order to do so, we determined a relative scaling factor between the maps from two different microscopes by iterative adjustment of the pixel size in one of the maps, followed by calculation of the correlation-coefficient in ChimeraX against the reference map (Wilkinson et al., 2019). As a result, a relative pixel size for the EMBL dataset was adjusted to 0.816 (instead of 0.810) and the CTF parameters were determined again for this dataset. To achieve the best possible scaling of this dataset against the ESRF reference (0.83 $Å \cdot pixel^{-1}$), we determined optimal box sizes for the extraction and the 786 k preselected ESRF particles

were re-extracted in a 348x348 pixel box, while preselected 570 k EMBL particles were re-extracted in a 354x354 pixel box, down-scaled to 348x348 pixels during the extraction. Both datasets were merged and the resulting 1.35 million particles were subjected to 2 rounds of 2D and one round of 3D classification. The best 3D class containing 541 k particles was refined to 4.9 Å resolution. Further 3D classification into 5 classes gave 2 equally good reconstructions, which differed in angular distribution. As previous data processing indicated a missing angle problem, both classes (312 k particles) were used in a subsequent 3D refinement yielding a reconstruction at 3.9 Å resolution. This reconstruction was used for CTF refinement and Bayesian polishing (Zivanov et al., 2019). Although we could not identify any significant heterogeneity in our data at this stage, further 3D classification allowed us to select a subset of particles, with presumably higher signal-to-noise ratio, which refined to 3.6 Å-resolution (from 99K particles) and 3.5 Å-resolution (from 27 k particles). The latter was used for subsequent model building.

The map used for *de novo* model building is missing some of the peripheral regions including the INTS9/11 CTD2 dimer and INTS4^{CTD}. While we were performing focused classification and refinement, we realized that classes containing well-defined peripheral regions originate almost exclusively in the ESRF1 dataset. It is possible that variation in sample preparation, vitrification conditions and/or ice thickness preserved those fragile elements only in 1 out of 3 datasets. Therefore, we performed focused classification and refinement on this dataset alone. Briefly, the initially picked 1.4 million particles were subjected to 3 rounds of 2D and 3 rounds of 3D classifications in order to remove broken particles and to select well-aligning classes. The resulting 115 k particles were refined to 4.4 Å-resolution. The angles assigned in this refinement were used for subsequent signal subtraction (Bai et al., 2015) followed by 3D classification without image alignment and T = 40. The best classes were reverted to the original particles and refined. Both maps yielded a final resolution of 6.5 Å.

All reported resolutions were calculated within Relion using gold standard Fourier Shell Correlation (FSC) procedures (Scheres and Chen, 2012).

Model Building and Refinement

For INTS9 and INTS11 homology models were calculated using the I-TASSER web server(Zhang, 2008) and fitted into the cryo-EM map in ChimeraX (Goddard et al., 2018). Based on the slightly larger molecular weight of INTS9, this subunit was assigned to the larger lobe, which was later confirmed by the amino acid register and identification of a unique insertion (NAD) in the INTS9^{MBL} domain. Homology models were rebuilt manually in Coot (0.8.9.2-pre EL) (Emsley and Cowtan, 2004). The density of the CTD1 dimer was good enough for *de novo* modeling. The CTD2 dimer was modeled by rigid-body docking of the previously reported coordinates (PDB ID: 5V8W) into a low-resolution lobe protruding from the end of the CTD1 dimer. Additionally, the linker helix connecting the CTD1 and CTD2 of INTS11 (495-504), which is as well part of the previously reported crystal structure of CTD2 (PDB ID: 5V8W), associates more tightly with the CTD1 dimer in our structure and its position is in agreement with the current CTD2 assignment.

The directionality of the INTS4 helical repeat was identified by crosslinking and mass spectrometry. The model for HEAT repeats 1-9 was built *de novo* into the high-resolution map and the register assignment was based on visible side-chain densities and secondary structure predictions (Kelley et al., 2015). The length of predicted helices is in good agreement with the visible density. Additionally, residues 264-280 are predicted to form a short extended loop between repeats H6 and H7, which is indeed visible in our map and provides a landmark verifying a correct sequence assignment.

A medium resolution map obtained from focused classification and refinement was used to extend the model with 3 additional HEAT repeats, whose register was tentatively assigned based on the secondary structure predictions.

The atomic model was refined in reciprocal space with Refmac5 (Murshudov et al., 2011) with the secondary structure restrains generated in ProSMART (Nicholls et al., 2014) within the CCP-EM (1.3.0) software suit (Burnley et al., 2017) . Half-map validation was performed as previously described (Brown et al., 2015). The atomic model was visualized in ChimeraX (Goddard et al., 2018) and PyMol and the electrostatic calculations were performed with the APBS plugin in PyMol (Schrödinger).

Cross-linking Mass spectrometry

Protein complexes were purified as previously described and adjusted to a concentration of 1 mg/ml in a 50 μL volume. DSS (disuccinimidyl suberate) was dissolved in Dimethylformamide with a final concentration of 50 mM. The dissolved crosslinker was added to the protein aliquot with a final concentration of 1 mM and 10 mM respectively and incubated at 35°C for 30 min. The reaction was quenched with 0.1 volumes of 1M Ammoniumbicarbonate and again incubated for 10 min at 35°C. Subsequently the sample was treated with 0.8 volumes of 10 M Urea and 250 mM Ammoniumbicarbonate as well as 0.05 volumes RapiGest SF Surfactant (Waters, cat. No. 186008090) and sonicated for 1 min in an ultrasound bath. Later, DTT was added with a final concentration of 10 mM, incubated for 30 min at 37°C and freshly prepared Iodoacetamide was added with a final concentration of 15 mM and again incubated for 30 min at room temperature (protected from light).

Subsequently, the sample was treated with different proteases beginning with Endoproteinase Lys-C (5 μL of an 0.1 mg/ml stock in Ammoniumbicarbonate) and incubated for 4 h at 37°C. The Urea concentration was adjusted to 1.5 M with HPLC grade water and Trypsin was added (1 μL of an 1 mg/ml stock) and incubated over night at 37°C. Subsequently the sample was acidified with Trifluoroacetic acid (1% v/v final concentration), incubated at 37°C for 30 min and spun down at 17,000 g for 5 min. The supernatant was discarded and the pellet was frozen in liquid nitrogen and stored at −80°C or dry ice. The mass spectrometry experiment and cross-links identification was performed by the EMBL Proteomics Core Facility in Heidelberg.

Analytical pull-down and quantitative mass spectrometry

The cell lines expressing Protein A – TEV – SBP N-terminal tagged INTS4, INTS5, INTS7, INTS10 or INTS14 were grown in 150 mL Freestyle media to a density of $1x10^6$ cells/ml and harvested at 300 g (JA14) for 10 min and 4°C. The cells were disrupted and fractionated following Dignam's nuclear extract preparation protocol (Dignam et al., 1983) Nuclear extract and S100 fractions were frozen in liquid nitrogen and stored at −80°C until further use. The S100 or the nuclear extract fractions were thawed, and incubated with IgG beads for 2 h on a turning wheel at 4°C. Subsequently the beads were washed with Buffer3 (150 mM KCl, 20 mM HEPES-KOH pH 7.8) and 200 μL Buffer3 containing 12.5ug of TEV protease was added to the IgG beads to elute the proteins. The digestion was performed at 20°C for 90 min and the resin was additionally eluted 4 times with 200 μL of Buffer3. The elution fractions were pooled, added to Steptavidin beads and incubated again for 90 min on a turning wheel at 4°C. Subsequently, the beads were washed and eluted in 70 μL Buffer4 (150 mM KCl, 20 mM HEPES-KOH pH 7.8, 10 mM desthiobiotin). The eluate was spun down at 17,000 g and 4°C or 5 min, the supernatant collected and flash frozen in liquid nitrogen and stored at −80°C or on dry ice until further use.

For the mass spectrometric analysis, 40 μL of SBP elution fractions obtained from nuclear extract (INTS4 and INTS5, shown in Figures 1B and 1C) or S100 fraction (INTS4 and INTS10, shown in Figures 1D and 1E) were subjected to an in-solution tryptic digest using a modified version of the Single-Pot Solid-Phase-enhanced Sample Preparation (SP3) protocol (Hughes et al., 2014; Moggridge et al., 2018). Samples were added to Sera-Mag Beads (Thermo Scientific, #4515-2105-050250, 6515-2105-050250) in 20 μl 15% formic acid and 60 μl of ethanol. Binding of proteins was achieved by shaking for 15 min at room temperature. SDS was removed by 4 subsequent washes with 200 μl of 70% ethanol. Proteins were digested with 0.4 μg of sequencing grade modified trypsin (Promega, #V5111) in 40 μl Na-HEPES, pH 8.4 in the presence of 1.25 mM TCEP and 5 mM chloroacetamide (Sigma-Aldrich, #C0267) overnight at room temperature. Beads were separated, washed with 10 μl of an aqueous solution of 2% DMSO and the combined eluates were dried down. Peptides were reconstituted in 10 μl of H_2O and reacted with 80 μg of TMT10plex (Werner et al., 2014) (Thermo Scientific, #90111) label reagent dissolved in 4 μl of acetonitrile for 1 h at room temperature. Excess TMT reagent was quenched by the addition of 4 μl of an aqueous solution of 5% hydroxylamine (Sigma, 438227). Peptides were mixed to achieve a 1:1 ratio across all TMT-channels. Mixed peptides were subjected to a reverse phase clean-up step (OASIS HLB 96-well μElution Plate, Waters #186001828BA) and analyzed by LC-MS/MS on a Q Exactive Plus (Thermo Scientific) as previously described (Becher et al., 2018).

Briefly, peptides were separated using an UltiMate 3000 RSLC (Thermo Scientific) equipped with a trapping cartridge (Precolumn; C18 PepMap 100, 5 lm, 300 lm i.d. × 5 mm, 100Å) and an analytical column (Waters nanoEase HSS C18 T3, 75 lm × 25 cm, 1.8 lm, 100Å). Solvent A: aqueous 0.1% formic acid; Solvent B: 0.1% formic acid in acetonitrile (all solvents were of LC-MS grade). Peptides were loaded on the trapping cartridge using solvent A for 3 min with a flow of 30 μl/min. Peptides were separated on the analytical column with a constant flow of 0.3 μl/min applying a 2 h gradient of 2 – 28% of solvent B in A, followed by an increase to 40% B. Peptides were directly analyzed in positive ion mode applying with a spray voltage of 2.3 kV and a capillary temperature of 320°C using a Nanospray-Flex ion source and a Pico-Tip Emitter 360 lm OD × 20 lm ID; 10 lm tip (New Objective). MS spectra with a mass range of 375–1.200 m/z were acquired in profile mode using a resolution of 70.000 [maximum fill time of 250 ms or a maximum of 3e6 ions (automatic gain control, AGC)]. Fragmentation was triggered for the top 10 peaks with charge 2–4 on the MS scan (data-dependent acquisition) with a 30 s dynamic exclusion window (normalized collision energy was 32). Precursors were isolated with a 0.7 m/z window and MS/MS spectra were acquired in profile mode with a resolution of 35,000 (maximum fill time of 120 ms or an AGC target of 2e5 ions).

Acquired data were analyzed using IsobarQuant (PMID: 26379230) and Mascot V2.4 (Matrix Science) using a reverse UniProt FASTA *Homo sapiens* database (UP000000589) including common contaminants. The following modifications were taken into account: Carbamidomethyl (C, fixed), TMT10plex (K, fixed), Acetyl (N-term, variable), Oxidation (M, variable) and TMT10plex (N-term, variable). The mass error tolerance for full scan MS spectra was set to 10 ppm and for MS/MS spectra to 0.02 Da. A maximum of 2 missed cleavages were allowed. A minimum of 2 unique peptides with a peptide length of at least seven amino acids and a false discovery rate below 0.01 were required on the peptide and protein level (Savitski et al., 2015).

The raw output files of IsobarQuant were processed using the R programming language (ISBN 3-900051-07-0). Only proteins that were quantified with at least two unique peptides were considered for the analysis. Raw signal-sums (signal_sum columns) were normalized using variance stabilization normalization (Huber et al., 2002). Ratios were computed using these normalized TMT reporter ion signals. The top3 value is the average log10 MS1 intensity of the three most abundant peptides for each protein and serves as an estimator for the average abundance of a protein in the multiplexed mass spec run.

The mass spectrometry experiment and data analysis was conducted by the EMBL Proteomic Core Facility in Heidelberg.

RNA *in vitro* transcription and labeling

RNA substrates for binding studies were generated by T7 run-off transcription using the Milligan transcription method from annealed template DNA oligonucleotides (Milligan et al., 1987). The U1 stem-loop 4 substrate comprises human U1 snRNA nucleotides 137-164 followed by 34 downstream nucleotides, including the 3′-box sequence. The control RNA is a scrambled sequence of the same size. Transcription products were gel purified, enzymatically capped with the vaccinia capping enzyme (NEB) following manufacturer recommendations and labeled with fluorescein at the 3′ end. Briefly, the 3′ vicinal diol was oxidized by re-suspending the RNA in 100 μL of freshly made oxidation solution (0.1 M sodium periodate and 0.1 M sodium acetate (pH 5.0)) and incubated at room

temperature for 1.5 h in the dark. The reaction was quenched by the addition of 10 μL of 3 M KCl, placed on ice for 10 min, and the resulting insoluble KIO_4 was pelleted by a brief centrifugation. A thiosemicarbazide derivative of fluorescein (100 mM in DMSO) was added to a final concentration of 50 mM and incubated at room temperature for 4 h, as previously described (Hardin et al., 2015). Labeled products were purified using a denaturing PAGE.

EMSA

Fluorescein labeled RNA was adjusted to a final concentration of 10 nM, mixed with a 2-fold dilution series of purified INTS4/9/11 with a maximum concentration of 10 μM and incubated on ice for 2h. Subsequently the sample was loaded onto a 1% TBE-Agarose gel and run for 120 min at 50 V and 4°C. Gels were visualized with the Chemidoc imaging system (Bio-Rad).

Dot blot

Dot-Blots were used to trace the crosslinked protein samples after the GraFix. A PVDF membrane was incubated for 5 min in PBS and installed on a Dot blot rack (BioRad) and washed twice with 100 μL PBS. 10 μL of the crosslinked and fractionized GraFix were mixed with 100 μL PBS and blotted onto the PVDF membrane. Subsequently, the membrane was washed twice with 100 μL PBS and transferred to 5% milk in PBST. After 1 h incubation the milk was replaced by Anti-SBP antibody (Merck, cat. MAB10764), 1:5000 dilution) in 5% milk in PBST. 1 hour later, the antibody was removed, the membrane washed twice for 5 min with PBST and incubated again for 1 h with goat Anti-Mouse-IgG-HRP conjugate (Thermo Fisher, 3cat. 1430, 1:2000 dilution). Subsequently, the membrane was washed 4 times with PBST, developed with Pirece^{TM} ECL Western Blotting Substrate (Thermo Fisher, cat. 32106, 1ml) and imaged with a Bio-Rad Chemidoc system.

GFP-based *in vivo* reporter assay

The reporter plasmid was cloned by inserting U7 snRNA gene (including 200 nt of the promoter and 70 nt of the downstream regions) into a modified backbone of the pFLAG_CMV10 vector (SIGMA) followed by EGFP ORF, based on the previously described design (Albrecht and Wagner, 2012).

Integrator subunit knock-downs were performed in HEK293T cells (ATCC) using Lipofectamine RNAiMAX (Invitrogen) and the following siRNAs: scrambled control (UGCACCGAGUGGCGACACCUU), INTS4 (GUAGGCUUAAGGAGUAUGUGAUU) and INTS11 (CAGACUUCCUGGACUGUGUUU) used in a previous study (Albrecht et al., 2018).

Integrator subunits for the rescue experiments were cloned into a modified pFLAG_CMV10 vector with N-terminal affinity tags: 3xFLAG for INTS11, 3xHA for INTS9 and SBP for INTS4. 3xFLAG_INTS11 was carrying silent mutations providing resistance to the siRNA treatment.

24 h before transfection, cells were seeded into 24-well plates to reach 60%–80% confluency at the time of transfection. For each condition 0.8 pmol of siRNA was diluted in 50 μL of opti-MEM and mixed with 50ul of opti-MEM containing 2.4ul of RNAi-MAX (Invitrogen). After 5 min incubation, the transfection mixture was added dropwise to each well, containing 500 μL of DMEM/10% FBS (GIBCO). After 24 h incubations the cells were split and re-seeded in a 1:3 ratio. After 24 h incubation the cells were transfected again with siRNA as described above. The following day, cells were split in 1:2 ratio, 12h before transfection of the reporter and rescue plasmids. For each well, 300 ng of the U7_GFP reporter plasmid was mixed with 50 ng of P2A-mCherry-N1 (Addgene #84329) and 300 ng of the rescue plasmids (or pFLAG_CMV10 negative control) in a total volume of 50 μL with DMEM (GIBCO), without FBS. The reporter/rescue/DMEM solution was mixed with 50 μL DMEM (no FBS) containing 1.95ul of LipoD293 (Sinagen). After 15 min incubation at room temperature, the mixture was added dropwise to each well.

Cells were harvested 36 h later by removing the media, adding 500 μL of cold PBS and gentle pipetting until the cells were detached. The harvested cells were spun down at 300 g for 2 min in a cooled table top centrifuge, resuspended in 50 ul RIPA buffer (150mM NaCl, 50mM Tris-Cl pH 8.0, 1%NP-40, 0.5% deoxycholate, 0.1% SDS) and transferred to a black, 96-well plate (Corning) for the fluorescence readout using a Clariostar plate reader.

The raw GFP fluorescence intensity (FI) was divided by mCherry FI (transfection control) and normalized against the mock-treated control. Each condition was done in biological triplicates. The error bars in the figures correspond to the standard deviation of the measurements.

After FI readout, samples were analyzed for transgene expression by western blot.

Immunoprecipitation assay

HEK293T cells were seeded into 6-well plates with a density of 500 000 cells per well in 1.5 mL D-MEM medium supplemented with 10% FBS. Cells were transfected 24 h later by preparing 50 μL Opti-MEM with 1 μg of each transfected plasmid and 50 μL Opti-MEM with 3 μg PEI25k per μg plasmid for each well. Subsequently, PEI and Plasmid were mixed, incubated at room temperature for 30 min and dropwise added into the cells. After 24 h of transfection 1.5 mL D-MEM/FBS was added to each well. 48 h after transfection the cells were harvested by removing the media, adding PBS and detaching the cells by gentle pipetting. The cells were spun down, resuspended in 400 μL lysis buffer (150 mM KCl, 20 mM HEPES-KOH pH 7.8 and 0.1% Triton X-100) and sonicated for 10 s at 30% amplitude. Next, the sonicated cells were spun down using a table top centrifuge at 20 000 g at 4°C for 30 min and the supernatant was added onto affinity resin (HA agarose) to capture the bait protein. The beads and the

lysate were incubated for 2h on a turning wheel. Subsequently, the beads were spun down, the supernatant removed and the beads were washed 3 times with 150 mM KCl and 20 mM HEPES-KOH pH 7.8. Finally, the beads were resuspended in SDS sample buffer and heated up to 85°C for 5 min to release bound proteins. Input and elution fractions were analyzed by western blotting.

Western blot

A PVDF membrane (Merck) was activated for 2 s in 100% EtOH and incubated for 5 min in transfer buffer (1xTris-Glycine, 20%EtOH). A wet transfer was performed for 60-90min at 30V in an Invitrogen blotting chamber. The membrane was blocked with 5% milk in PBS supplemented with 0.1% Tween 20 (hereafter referred to as PBST) for 1h at room temperature. Primary antibodies were added (anti-INTS4 - 1:2000; anti-FLAG – 1:5000; anti-GAPDH – 1:10000, anti-HA – 1:5000; all antibodies were diluted in 5% milk with PBST) and incubated for 1h at room temperature. The membrane was washed 3 times for 5 min with PBST and in case of INTS4 or GAPDH detection anti-rabbit IgG (1:10 000 in 5% milk in PBST) was added and incubated at room temperature for 1h. The membrane was washed 3 times for 5min with PBST and imaged using chemiluminescent substrate detection based on HRP (Pierce) in a Chemidoc imager (Bio-Rad).

QUANTIFICATION AND STATISTICAL ANALYSIS

The error bars in Figures 3 and 6 correspond to the standard deviation of the 3 individual measurements.

For the EMSA assay (Figures 6 and S1), individual bands were quantified in Image lab (Bio-Rad) to calculate fraction of RNA bound by protein in each condition. The titration curves were modeled using a modified Hill equation, as previously described (Ryder et al., 2008). The uncertainty of the apparent Kd estimation (Figure S6) was estimated based on standard deviation of the nearest experimental titration point.

Molecular Cell, Volume *81*

Supplemental information

Structure of the catalytic core of the Integrator complex

Moritz M. Pfleiderer and Wojciech P. Galej

SUPPLEMENTARY INFORMATION

Figure S1

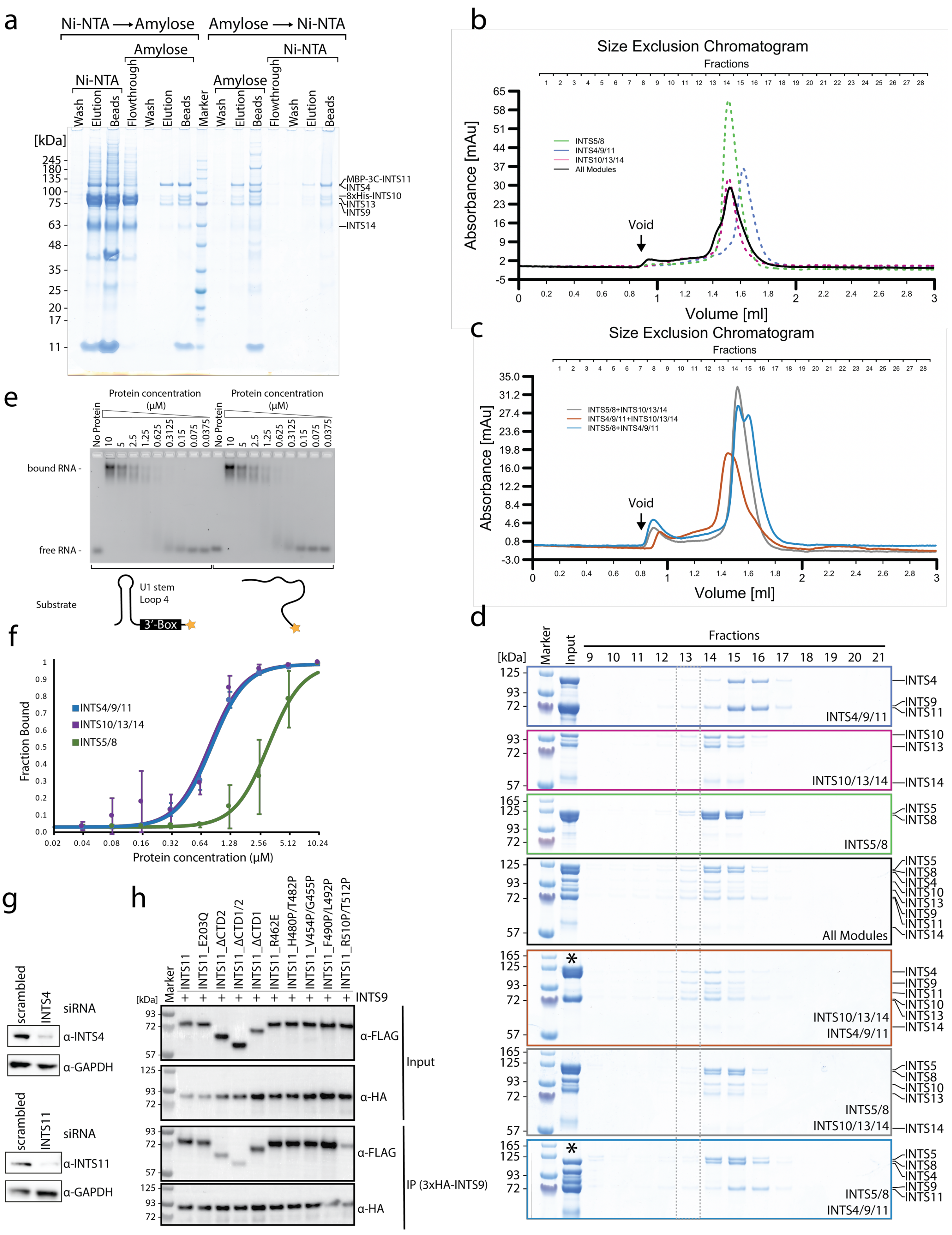

Figure S1. Higher order complex formation by INTS4/9/11 and INTS10/13/14 and their RNA-binding properties. Related to Figures 1,3,4 and 6.

a, SDS-PAGE showing the results of recombinant expression and tandem affinity purification of all 6 Integrator subunits forming INTS4/9/11 and INTS10/13/14 modules. MBP tag was fused to INTS11 and 6xHistag was attached to INTS10. **b,** SEC profile showing the elution volume of INTS5/8, INTS4/9/11 and INTS10/13/14 as single complexes (dotted lines) and after being mixed together (black line). **c,** SEC profiles of the different sub-complexes mixed together to test their interactions with one another. **d,** SDS-PAGE of the different fractions corresponding to the peaks of the SECs in panel b and c. Dotted line indicates fractions containing the higher order complex. Two lanes marked with asterisks (*) were accidentally swapped during SDS-PAGE loading. **e,** EMSA titration experiment, in which all 3 modules were used to assess their RNA binding. No clear cooperativity was observed when compared to individual titrations (Figure 6) **f,** Semi-quantitative RNA-binding analysis based on EMSA experiments performed in triplicates. The titration curves report the fraction of RNA bound ([RNP]/[RNA]+[RNP]) against protein concentration (logarithmic scale). The estimated apparent Kd's are as follows: 0.82±0.06 μM (INTS4/9/11) 3.1±1 μM (INTS5/8) and 0.78±0.08 μM (INTS10/13/14). The uncertainty of the apparent Kd was estimated based on standard deviation of the nearest experimental titration point. **g,** Western blots showing efficiency of the siRNA knock-down experiment for INTS4 and INTS11. **h,** Western blot showing results of the HA-agarose pull-down experiments from HEK293T cells co-expressing 3xHA-INTS9 and 3xFLAG-INTS11 variants.

Figure S2

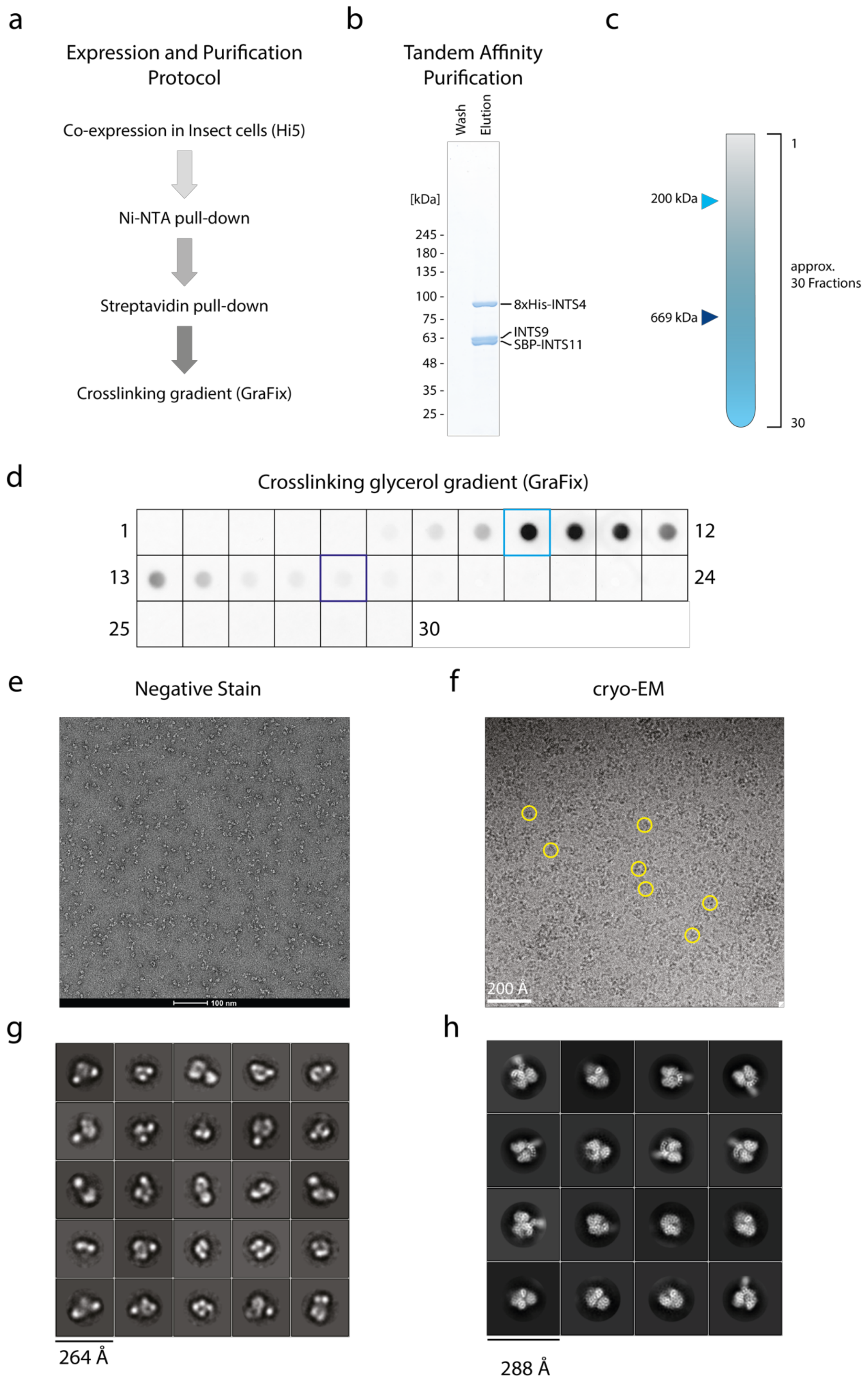
a
Expression and Purification Protocol
Co-expression in Insect cells (Hi5)
Ni-NTA pull-down
Streptavidin pull-down
Crosslinking gradient (GraFix)
b
Tandem Affinity Purification
Wash
Elution
[kDa]
245 -
180 -
135 -
100 -
75 -
63 -
48 -
35 -
25 -
8xHis-INTS4
INTS9
SBP-INTS11
c
200 kDa
669 kDa
1
approx. 30 Fractions
30
d
Crosslinking glycerol gradient (GraFix)
1
12
13
24
25
30
e
Negative Stain
100 nm
f
cryo-EM
200 Å
g
264 Å
h
288 Å

Figure S2. Sample preparation and initial cryo-EM analysis. Related to Figure 2.

a, Flow chart of the expression and purification of the INTS4/9/11 complex. **b,** SDS-PAGE after the two-step purification indicated in panel a. **c** and **d,** Illustration of a calibrated cross-linking gradient and Dot-blot analysis of the different GraFix fractions with anti-SBP antibodies to detect the migration of the INTS4/9/11 complex. **e,** representative uranyl acetate negative stain micrograph. **f,** representative cryo-EM micrograph from a 300kV Titan Krios Microscope and K2 detector at -2.85 μm defocus. Examples of particles picked for processing are indicated with yellow circles. **g,** reference-free 2D class averages of a small negative stain dataset. **h,** reference-free 2D class averages of the cryo-EM dataset.

Figure S3

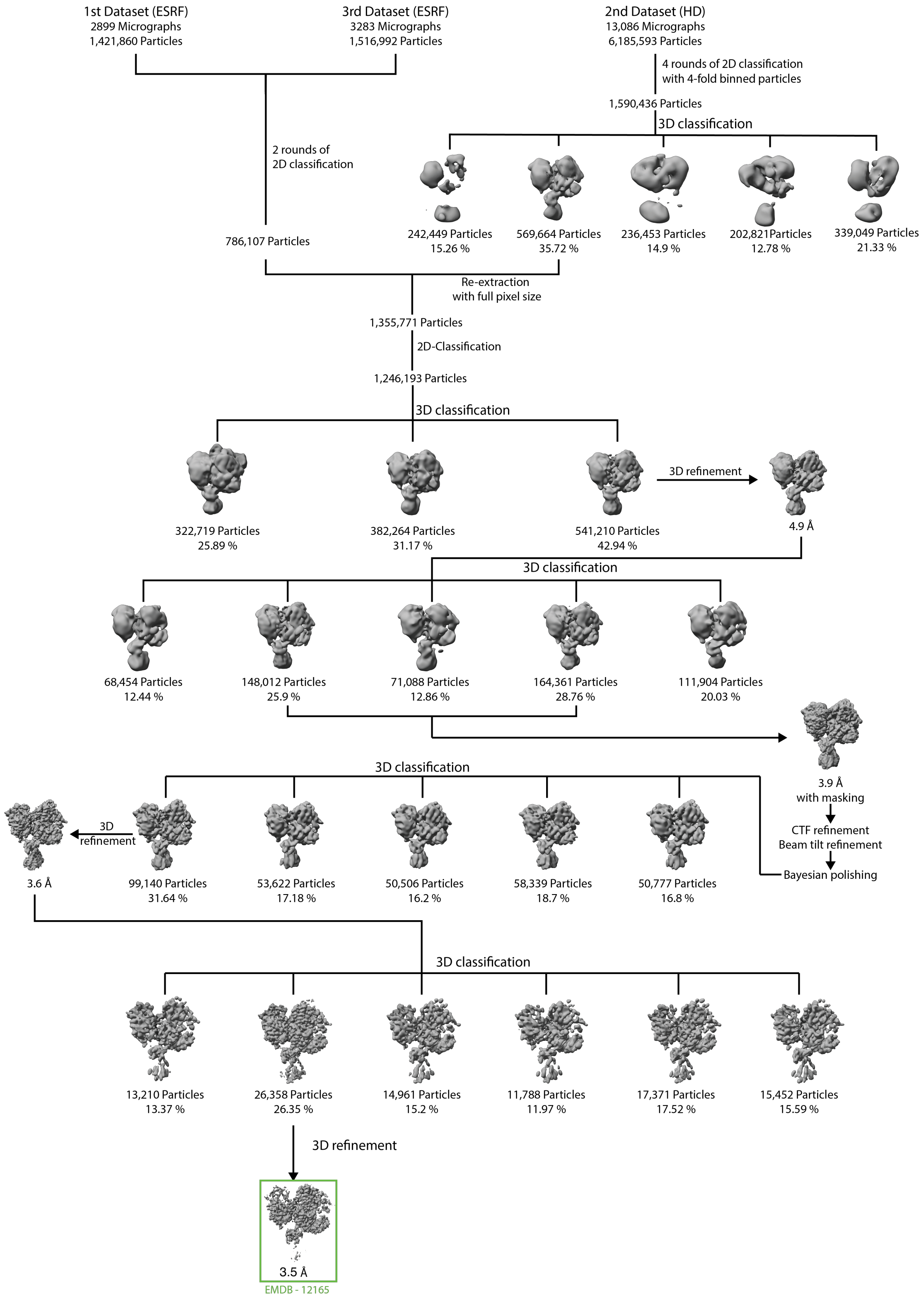

Figure S3. Workflow for the cryo-EM data processing. Related to Figure 2 and the STAR method section: Cryo-EM data collection and processing.

Three datasets were merged and processed as described in details in the method section.

Figure S4

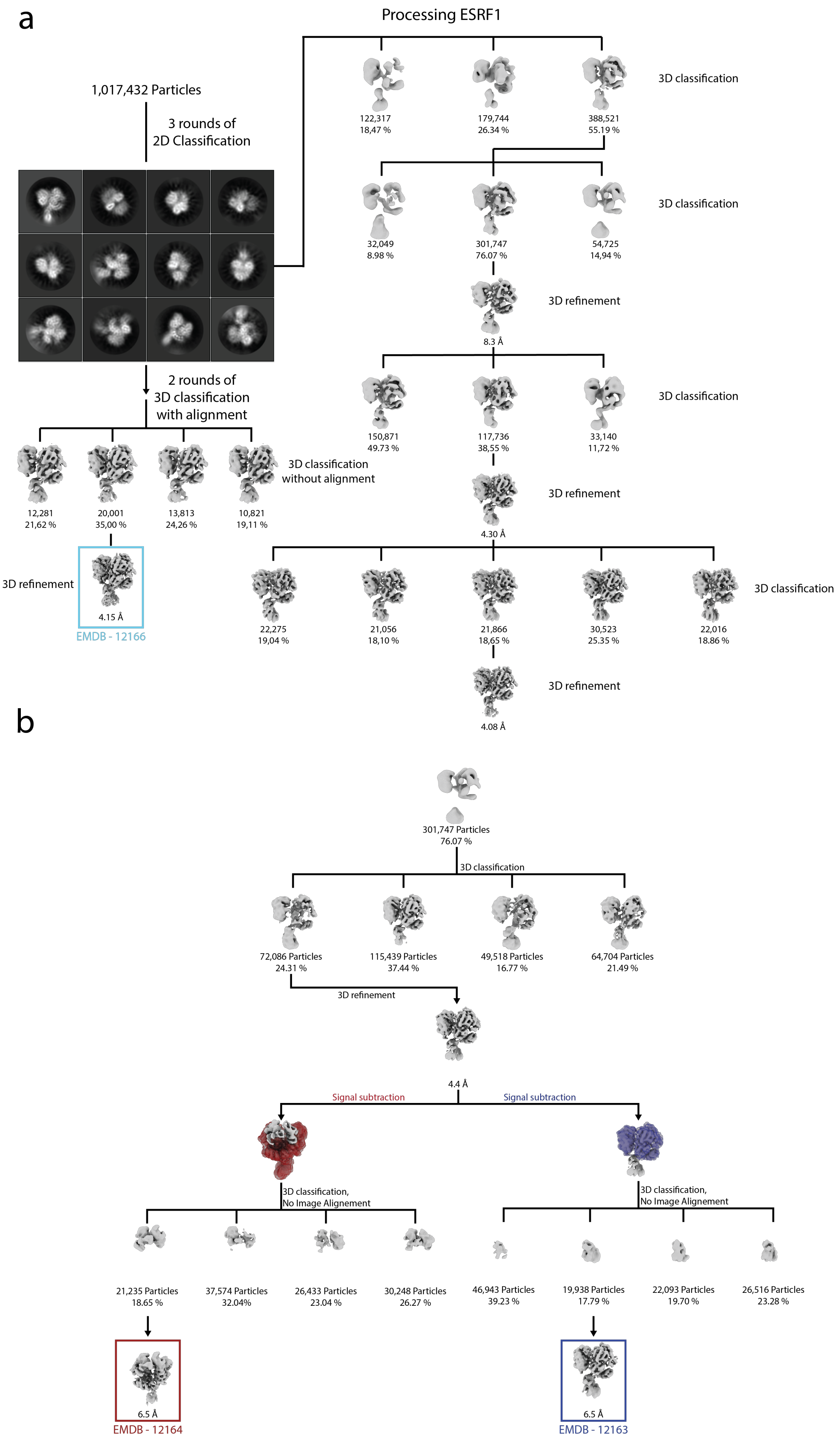
a
Processing ESRF1
1,017,432 Particles
3 rounds of
2D Classification
3D classification
122,317
18,47 %
179,744
26.34 %
388,521
55.19 %
3D classification
32,049
8.98 %
301,747
76.07 %
54,725
14,94 %
3D refinement
8.3 Å
3D classification
150,871
49.73 %
117,736
38,55 %
33,140
11,72 %
3D refinement
4.30 Å
2 rounds of
3D classification
with alignment
3D classification
without alignment
12,281
21,62 %
20,001
35,00 %
13,813
24,26 %
10,821
19,11 %
3D refinement
4.15 Å
EMDB - 12166
22,275
19,04 %
21,056
18,10 %
21,866
18,65 %
30,523
25.35 %
22,016
18.86 %
3D classification
3D refinement
4.08 Å
b
301,747 Particles
76.07 %
3D classification
72,086 Particles
24.31 %
115,439 Particles
37.44 %
49,518 Particles
16.77 %
64,704 Particles
21.49 %
3D refinement
4.4 Å
Signal subtraction
Signal subtraction
3D classification,
No Image Alignement
3D classification,
No Image Alignement
21,235 Particles
18.65 %
37,574 Particles
32.04%
26,433 Particles
23.04 %
30,248 Particles
26.27 %
46,943 Particles
39.23 %
19,938 Particles
17.79 %
22,093 Particles
19.70 %
26,516 Particles
23.28 %
6.5 Å
EMDB - 12164
6.5 Å
EMDB - 12163

Figure S4. Workflow for the focused classification and refinements used in this study. Related to Figure 2 and the STAR method section: Cryo-EM data collection and processing.

a, processing flowchart of the ESRF1 dataset contining peripheral regions of the complex. **b,** focussed classification and refinement workflow for $INTS4^{NTD}$ (red) and CTD2 (blue), based on ESRF1 dataset.

Figure S5

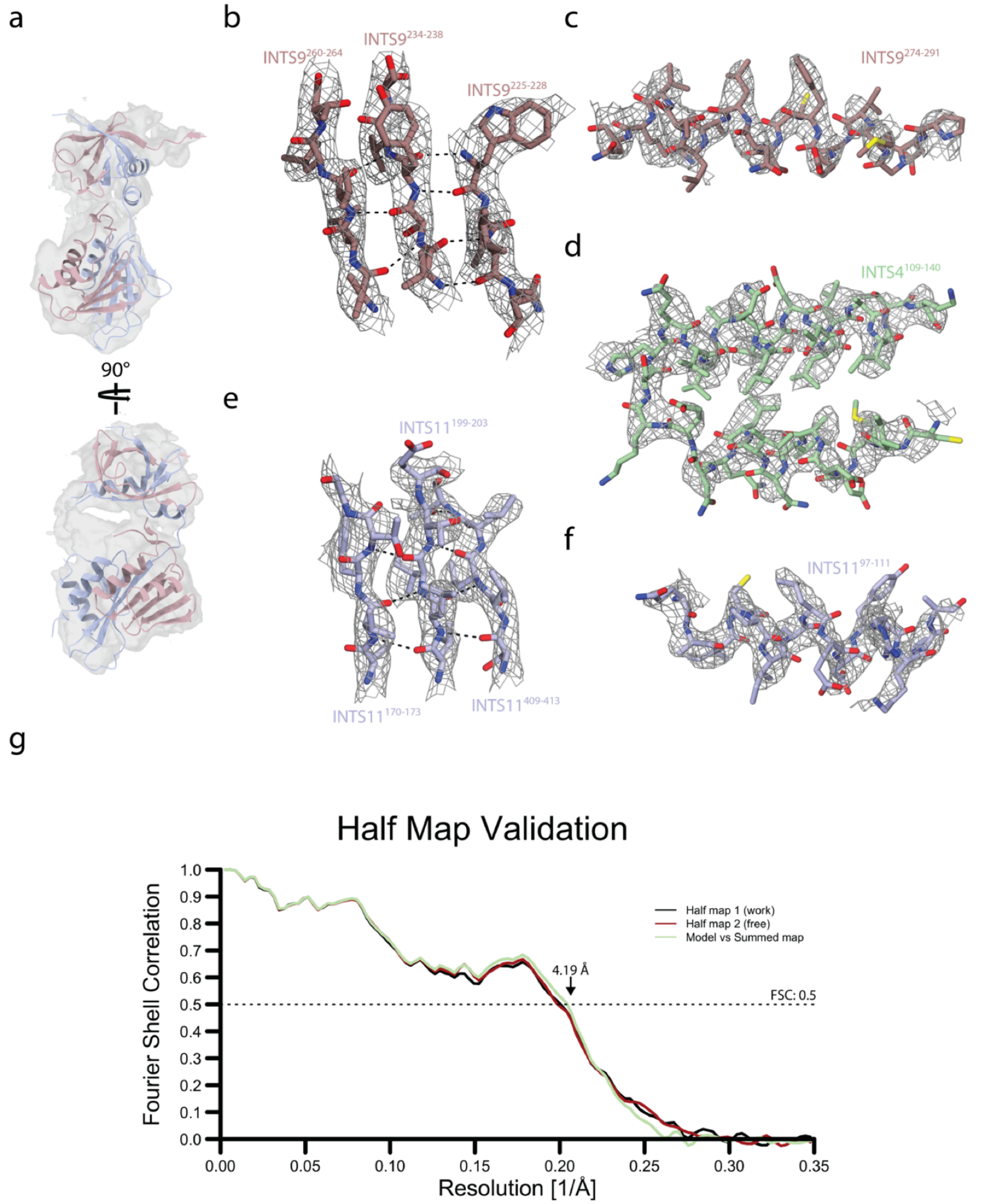

Figure S5. Model building of INTS4/9/11. Related to Figure 2.

a, INTS9/11^{CTD2}-focused map with the fitted crystal structure (PDB: 5V8W) and the de-novo modelled CTD1. **b-f,** Density at 3.5 Å with the built model of INTS4/9/11 in different areas of the map. **g,** Half map validation plot.

Figure S6

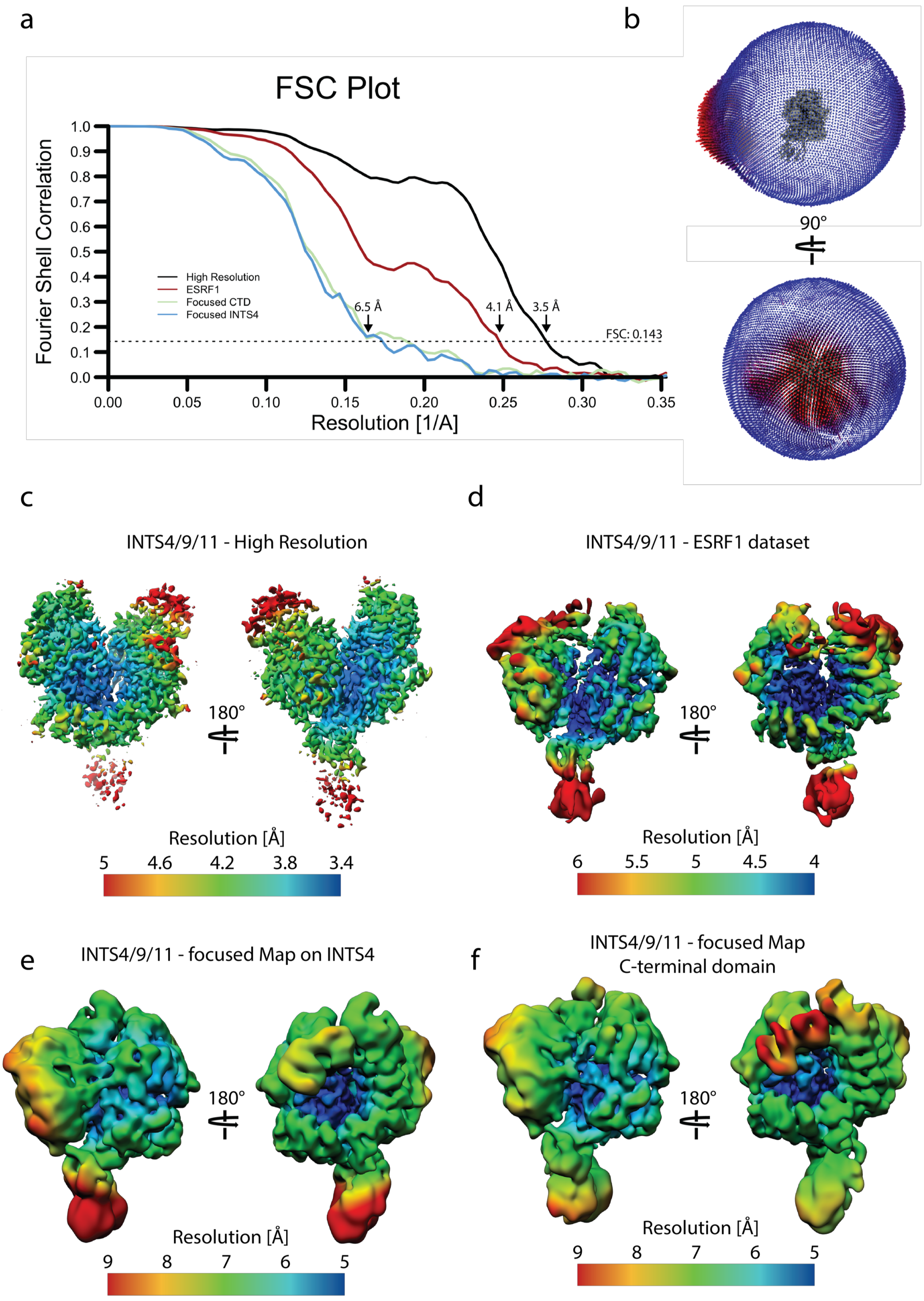

Figure S6. Global and local resolution of the different INTS4/9/11 maps generated in this study. Related to Figures 2, 3 and 4.

a, FSC plot for all four models used in this publication. **b,** Orientation distribution of the final, 3.5 Å map used for model building. **c-f,** cryo-EM maps from different reconstructions, coloured according to the local resolution.

Figure S7

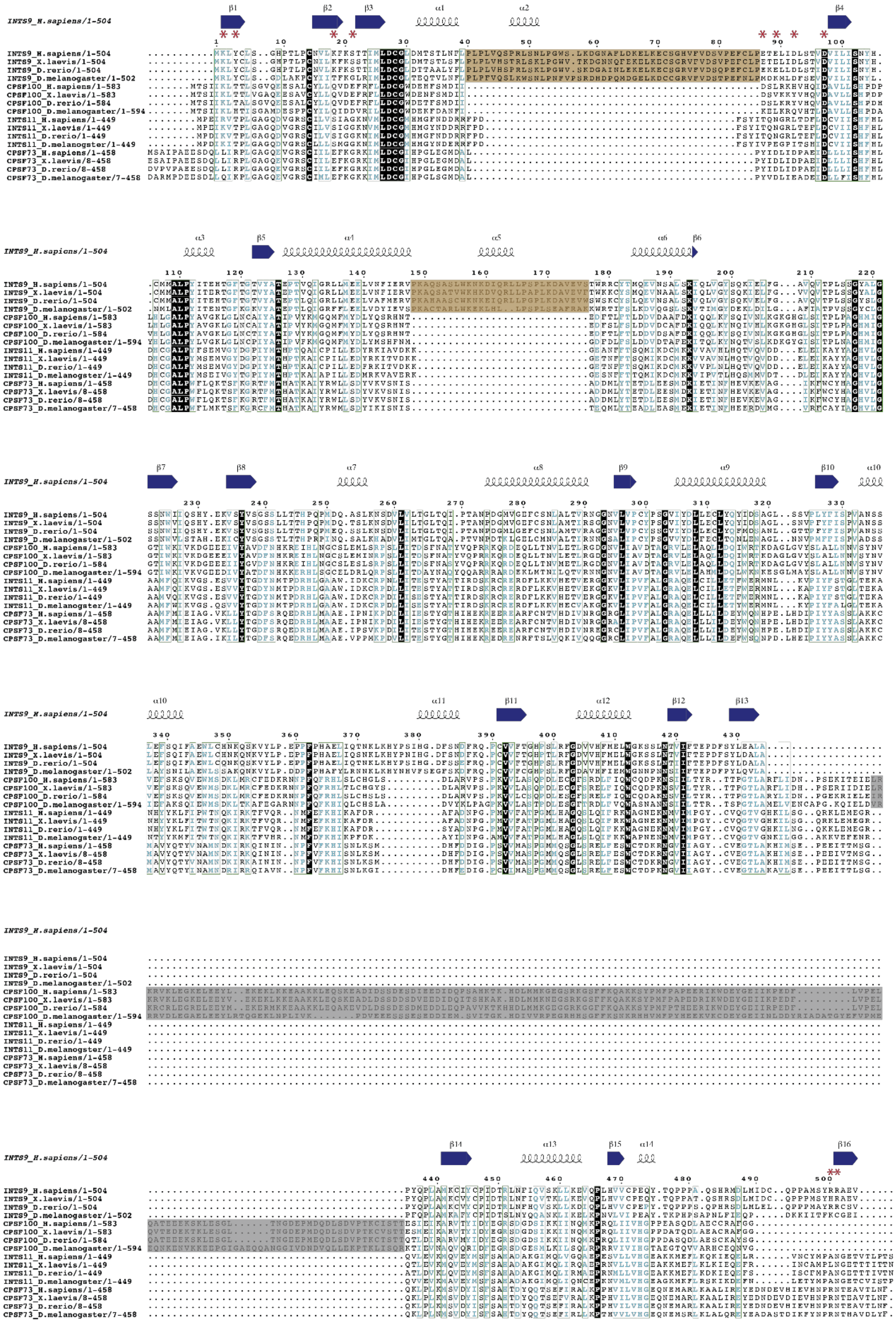

Figure S7. Comparison of the nuclease domains of INTS9, INTS11, CPSF73 and CPSF100. Related to Figures 2 and 5.

Structure-based alignment of INTS9, INTS11 CPSF73 and CPSF100 of different organisms (human - *H. sapiens*; African clawed frog – *X. laevis*; Zebrafish – *D. rerio*; Fruit fly – *D. melanogaster*). The NAD domain of INTS9 is highlighted in yellow and the disordered insert of CPSF100, which contains the PIM, is coloured in grey. Asterisks indicate the residues of the active centre of INTS11. Black background – strictly identical; light blue – conserved residues; green frame – conserved area.
